# In vitro metabolism of cathinone positional isomers: does sex matter?

**DOI:** 10.1007/s00216-023-04815-3

**Published:** 2023-07-15

**Authors:** Peng Che, J. Tyler Davidson, Kristina Still, Jeroen Kool, Isabelle Kohler

**Affiliations:** 1 Division of Bioanalytical Chemistry, Department of Chemistry and Pharmaceutical Sciences, Amsterdam Institute of Molecular and Life Sciences (AIMMS), Vrije Universiteit Amsterdam, De Boelelaan 1085, 1081 HV Amsterdam, The Netherlands; 2 Center for Analytical Sciences Amsterdam (CASA), Amsterdam, The Netherlands; 3 Department of Forensic Science, Sam Houston State University, Huntsville, TX USA; 4 Co van Ledden Hulsebosch Center (CLHC), Amsterdam Center for Forensic Science and Medicine, Amsterdam, The Netherlands

**Keywords:** Synthetic cathinones, Positional isomers, In vitro metabolism, Sex-specific differences, Methylmethcathinones

## Abstract

Synthetic cathinones, one of the most prevalent categories of new psychoactive substances, have been posing a serious threat to public health. Methylmethcathinones (MMCs), notably 3-MMC, have seen an alarming increase in their use in the last decade. The metabolism and toxicology of a large majority of synthetic cathinones, including 3-MMC and 2-MMC, remain unknown. Traditionally, male-derived liver materials have been used as in vitro metabolic incubations to investigate the metabolism of xenobiotics, including MMCs. Therefore, little is known about the metabolism in female-derived in vitro models and the potential sex-specific differences in biotransformation. In this study, the metabolism of 2-MMC, 3-MMC, and 4-MMC was investigated using female rat and human liver microsomal incubations, as well as male rat and human liver microsomal incubations. A total of 25 phase I metabolites of MMCs were detected and tentatively identified using liquid chromatography-tandem mass spectrometry (LC-MS/MS). Seven sex-specific metabolites were detected exclusively using pooled male rat liver microsomal incubations. In addition, the metabolites generated from the sex-dependent in vitro metabolic incubations that were present in both male and female rat liver microsomal incubations showed differences in relative abundance. Yet, neither sex-specific metabolites nor significant differences in relative abundance were observed from pooled human liver microsomal incubations. This is the first study to report the phase I metabolic pathways of MMCs using in vitro metabolic incubations for both male and female liver microsomes, and the relative abundance of the metabolites observed from each sex.

**Graphical abstract:**

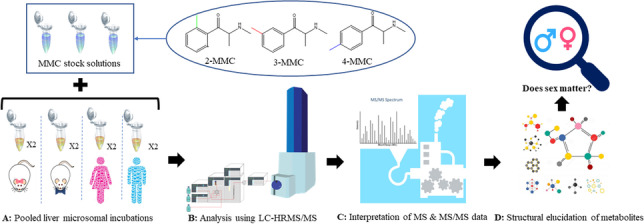

**Supplementary Information:**

The online version contains supplementary material available at 10.1007/s00216-023-04815-3.

## Introduction

New psychoactive substances (NPS), also referred to as designer drugs or “legal highs,” are defined as new narcotic/psychotropic drugs which are not controlled by the United Nations’ 1961 Narcotic Drugs and the 1971 Psychotropic Substances Conventions. These synthetic alternatives to conventional illicit drugs can pose a significant threat to the public health [1, 2]. Indeed, the NPS market is dynamic, with multiple new substances emerging each year, leading to challenges in monitoring and controlling these substances. Besides, the little information available on their pharmacology and toxicology can result in harmful consequences [2, 3]. Since most NPS are typically not controlled, they are easily available online or on the street market under the names “research chemicals”, “bath salts”, or “plant food” [4–6]. In the last decades, the category of synthetic cathinones (SCs) has seen a rise in its popularity worldwide, particularly among the young population [5, 6]. In 2020, SCs accounted for 65% of NPS material seized in Europe. Moreover, they currently represent the second largest category of NPS in terms of number of substances [7, 8]. SCs show cocaine- and amphetamine-like effects. Desired effects include increased energy, empathy, openness, and increased libido [9]. Both cardiovascular and neurological side effects have been reported, which are related to a decrease in the reuptake of norepinephrine, dopamine, and serotonin [10], as well as an increased release of dopamine [11]. Among all SCs, methylmethcathinone (MMC) isomers have been very popular among drug users, especially 3-methylmethcathinone (3-MMC) and 4-methylmethcathinone (4-MMC) [12, 13].

4-MMC, also known as mephedrone, has similar pharmacological effects to 3,4-methylenedioxymethamphetamine (MDMA, ecstasy) but with stronger craving feelings [14]. Due to its rapid increase in consumption and associated toxicology, 4-MMC has been banned in most European countries since 2010 [15]. As a consequence, its structural derivatives, such as 3-MMC, rapidly emerged on the drug market to replace 4-MMC [16, 17]. Due to an increase in the adverse events reports for 3-MMC worldwide and especially in Europe, the substance has been recently placed on the list of NPS under intensive monitoring by the European Monitoring Centre for Drugs and Drug Addiction (EMCDDA) [12]. The Netherlands currently represents an active player in NPS production, sale, and distribution within Europe, with 18 kg of 3-MMC powder shipped to European countries in 2020 [12]. The Dutch Poisons Information Center (DPIC) had seen a significant rise in the number of 3-MMC intoxication cases in 2020 and 2021 [13]. Due to multiple adverse events reported in the Netherlands and a growing concern, 3-MMC has been placed on the List II of the Opium Act in October 2021. As of now, more than 20 European countries have reported that 3-MMC is subject to restrictive measures at their national level [12]. Although 2-methylmethcathinone (2-MMC) has been less popular than other SCs, the ban of 3-MMC may shift the attention of drug users to other currently legal alternatives, such as 2-MMC.

The metabolism and toxicology of 4-MMC have already been investigated [18–24]. However, the metabolism of its positional isomers 3-MMC and 2-MMC remains mostly unknown. Moreover, little is known about the possible activity and/or toxicity of their metabolites. Information on drug metabolism is essential for forensic and toxicological purposes [25]. Due to ethical considerations, the investigation of NPS biotransformation is generally carried out using in vitro models, such as rat liver microsomes [26], human liver microsomes [21, 25, 27, 28], human hepatocytes [29], human S9 fractions [25, 30], or HepaRG cells [30]. These biological materials for in vitro incubations all contain drug-metabolizing enzymes, such as the cytochrome P450s (CYPs) complex.

Traditionally, metabolism studies are performed using male-derived materials. Indeed, women/females have been often excluded from (pre-)clinical trials and (pre-)clinical research investigating the effects and metabolism of drugs, regardless of the fact that substantial differences in pharmacokinetics and pharmacodynamics of drugs may exist between males and females, depending on the substance [31–33]. Until now, literature regarding potential sex-specific effects and metabolism of NPS in the organism remains very limited [34], even though sex-specific differences in pharmacokinetics and pharmacodynamics have been demonstrated for traditional recreational drugs, such as cannabinoids, cocaine, and morphine [35–37]. To the best of our knowledge, no study has investigated the effects of sex on the in vitro metabolism of SCs.

The objectives of this study are to identify the metabolites of MMCs using in vitro metabolic incubations and investigate potential sex-specific differences in MMCs biotransformation. The metabolites produced in in vitro models were analyzed and identified using liquid chromatography-tandem mass spectrometry (LC-MS/MS). As a confirmatory analysis, ultra-high pressure liquid chromatography combined with high-resolution mass spectrometry (UHPLC-HRMS) was used to further decipher metabolites that were co-eluting using conventional LC-MS/MS analysis. The identification of MMCs metabolites opens new avenues in clinical and forensic toxicology, by providing a better understanding of SC metabolism and helping with the interpretation of general unknown screening data. Moreover, the results highlight the importance of investigating sex-specific differences in NPS metabolism and risk assessment for clinical and forensic purposes.

## Material and methods

### Chemicals

Acetonitrile (LC-MS grade) and DMSO were purchased from Biosolve (Valkenswaard, The Netherlands). Formic acid (LC-MS grade) was obtained from Merck (Zwijndrecht, The Netherlands). Ultra-pure water was obtained from a Milli-Q Plus purification system (Millipore, Amsterdam, The Netherlands). Magnesium chloride, NADPH, glucose-6-phosphate, and glucose-6-phosphate dehydrogenase were purchased from Sigma (Zwijndrecht, The Netherlands). Pooled male rat (Sprague-Dawley) liver microsomes and pooled female rat (Sprague-Dawley) liver microsomes were obtained from Sigma-Aldrich (St. Louis, MO, USA). Pooled male human liver microsomes (Lot No. 2110108) and pooled female human liver microsomes (Lot No. 1210079) were obtained from XenoTech (Lenexa, Kansas City, KS, USA). 2-Methylmethcathinone (2-MMC) hydrochloride, 3-methylmethcathinone (3-MMC) hydrochloride, and 4-methylmethcathinone (4-MMC) hydrochloride standards were provided by the Amsterdam Dutch Police. All stock solutions were prepared in DMSO and stored at  -80 °C until further use.

### Pooled liver microsomal incubations

In order to investigate the biotransformation of the MMC positional isomers, the phase I metabolism of 4-MMC, 3-MMC, and 2-MMC was simulated in vitro using pooled human and rat liver microsomes. Specifically, each positional isomer was metabolized using pooled female human liver microsomes (pFHLM), pooled male human liver microsomes (pMHLM), pooled female rat liver microsomes (pFRLM), and pooled male rat liver microsomes (pMRLM). Each liver microsomal incubation was carried out in duplicate with a final volume of 100 µL. The reaction mixture consisted of 2.5 mM MgCl_2_ in 0.1 M potassium phosphate buffer (pH 7.4) containing 10% pooled liver microsomes [38]. Each drug was used at a concentration of 100 µM for all incubations. The incubations were initiated by addition of a NADPH regenerating system, resulting in a final concentration of 0.1 mM NADPH, 0.3 mM glucose-6-phosphate, and 0.4 U/mL glucose-6-phosphate dehydrogenase. The metabolic incubations took place at 37 °C for 60 min. The incubations were quenched with the addition of ice-cold acetonitrile, with a volume corresponding to twice the metabolic mixture volume, followed by centrifugation at 13,760 × *g* for 10 min at 4 °C. The supernatant was transferred to a new tube and evaporated with a vacuum-centrifuge until dryness. Prior to injection into the LC-UV or LC-MS/MS system, the dried residue was first dissolved with 40 µL eluent B, followed by the addition of 60 µL eluent A for reconstitution. Blank samples consisted of only DMSO without analyte; control samples were prepared without the addition of NADPH.

### Analysis of metabolic mixtures using liquid chromatography

The analysis of liver microsomal incubation mixtures was carried out using an Agilent 1260 Infinity II LC system (Waldbronn, Germany) equipped with an Agilent 1260 Infinity II Flexible Pump (G7104C), an Agilent 1260 Infinity II Variable Wavelength Detector (G7114A), an Agilent 1260 Infinity II Multicolumn Thermostat (G7116A), and an Agilent 1260 Infinity II Multisampler (G7167AA). The separation was carried out using an XBridge C_18_ column (4.6 mm × 100 mm, 3.5 μm particle size). The column and autosampler temperatures were set as 30 °C and 4 °C, respectively. The injection volume was 20 μL. The mobile phase was composed of water-acetonitrile-formic acid (97.9:2:0.1, *v/v/v*, eluent A) and acetonitrile-water-formic acid (97.9:2:0.1, *v/v/v*, eluent B). The separation gradient was the following: eluent B at 1% for 10 min, linear increase to 95% for 14 min, and isocratic step at 95% for 5 min. After each run, the column was re-equilibrated with the initial conditions for 5 min. The separation was performed at a flow rate of 0.6 mL/min. UV detection was performed at 254 nm and 280 nm.

### Metabolite identification using liquid chromatography-tandem mass spectrometry

Experiments for the identification of MMC metabolites were carried out using an Agilent 1260 Infinity II Analytical LC system connected to a maXis ultra-high resolution time-of-flight mass spectrometer (UHR TOF/MS, Bruker Daltonics, Bremen, Germany) via an electrospray ionization (ESI) source. To achieve a stable electrospray ionization (ESI), a flow splitter was used between the LC system and MS system, allowing 10% of the flow to enter the ESI source. The ESI source was operated in positive ionization mode. The capillary voltage and end plate offset were set at 4500 V and 500 V, respectively. The pressure of the nebulizer gas (nitrogen) was set to 4 bar. The temperature and flow rate of the drying gas (nitrogen) were 220 °C and 4.0 L/min, respectively. The quadrupole was set in scan mode with a mass range from *m/z* 50 to 400 at a spectral rate of 1 Hz. The AutoMS/MS mode was applied, where the five precursor ions from each cycle showing the highest intensity were selected for fragmentation. The product ions of MMCs were detected within a mass range of *m/z* 50 to 400 in the TOF/MS. Additional MS and MS/MS parameters are detailed in Supplementary Table S1. For metabolite identification, raw acquisition data were processed using the DataAnalysis software package from Bruker and the Elemental Composition Calculator v1.0 (written by Jef Rozenski, 1999, http://rna.rega.kuleuven.be/masspec/elcomp.htm), using 5 mDa mass tolerance for precursor and product ions. The instrument was externally calibrated using a 5 mmol L^−1^ sodium formate clusters tune mix.

### Enhancing the confidence in metabolite identification using ultra-high pressure liquid chromatography-tandem mass spectrometry

In order to enhance the confidence in metabolite identification and explain the presence of controversial product ions in the MS/MS spectrum for some metabolites due to co-eluting compounds, the metabolic mixtures of cathinone positional isomers were also analyzed using an Agilent 1290 Infinity II Analytical UHPLC system (Waldbronn, Germany) connected to a SCIEX ZenoTOF 7600 time-of-flight mass spectrometer (SCIEX, Singapore). The UHPLC system was equipped with an Agilent 1290 Infinity II High Speed Pump (G7120A), an Agilent 1290 Infinity II Multicolumn Thermostat (G7116B), and an Agilent 1290 Infinity II Vialsampler (G7129B). The separation was carried out using a Phenomenex Luna Omega C_18_ column (2.1 × 100 mm, 1.6 μm particle size) protected by a Phenomenex C_18_ guard column. The column and autosampler temperatures were set as 35 °C and 4 °C, respectively. The injection volume was 10 μL. The mobile phase was composed of water-formic acid (99.9:0.1, *v/v*, eluent A) and acetonitrile-formic acid (99.9:0.1, *v/v*, eluent B). The separation gradient was the following: 0–2 min 2% eluent B, 3–6 min linear increase to 10% eluent B, 6–7 min isocratic step at 10% eluent B, 7–11 min linear increase to 10% eluent B, 11–12 min isocratic step at 20% eluent B, 12–14 min a steep increase to 95% eluent B, 14–15 min isocratic at 95% eluent B, 15–16 min return to initial conditions of 2% eluent B and equilibration for 1 min. The separation was performed at a flow rate of 0.6 mL/min.

The ESI source was operated in positive ionization mode and parameters were set as the following: spray voltage, 5.5 kV; source temperature, 500 °C; nebulizer gas pressure, 45 psi; drying gas pressure, 45 psi; curtain gas pressure, 30 psi; and CAD gas pressure, 7 psi. Additional conditions are detailed in Supplementary Table S2. Raw acquisition data were processed using the SciexOS software from SCIEX and the Elemental Composition Calculator v1.0 (written by Jef Rozenski, 1999, http://rna.rega.kuleuven.be/masspec/elcomp.htm), using 5 mDa mass tolerance for precursor and product ions.

## Results and discussion

### MS/MS fragmentation of cathinone positional isomers

**Fig. 1:**
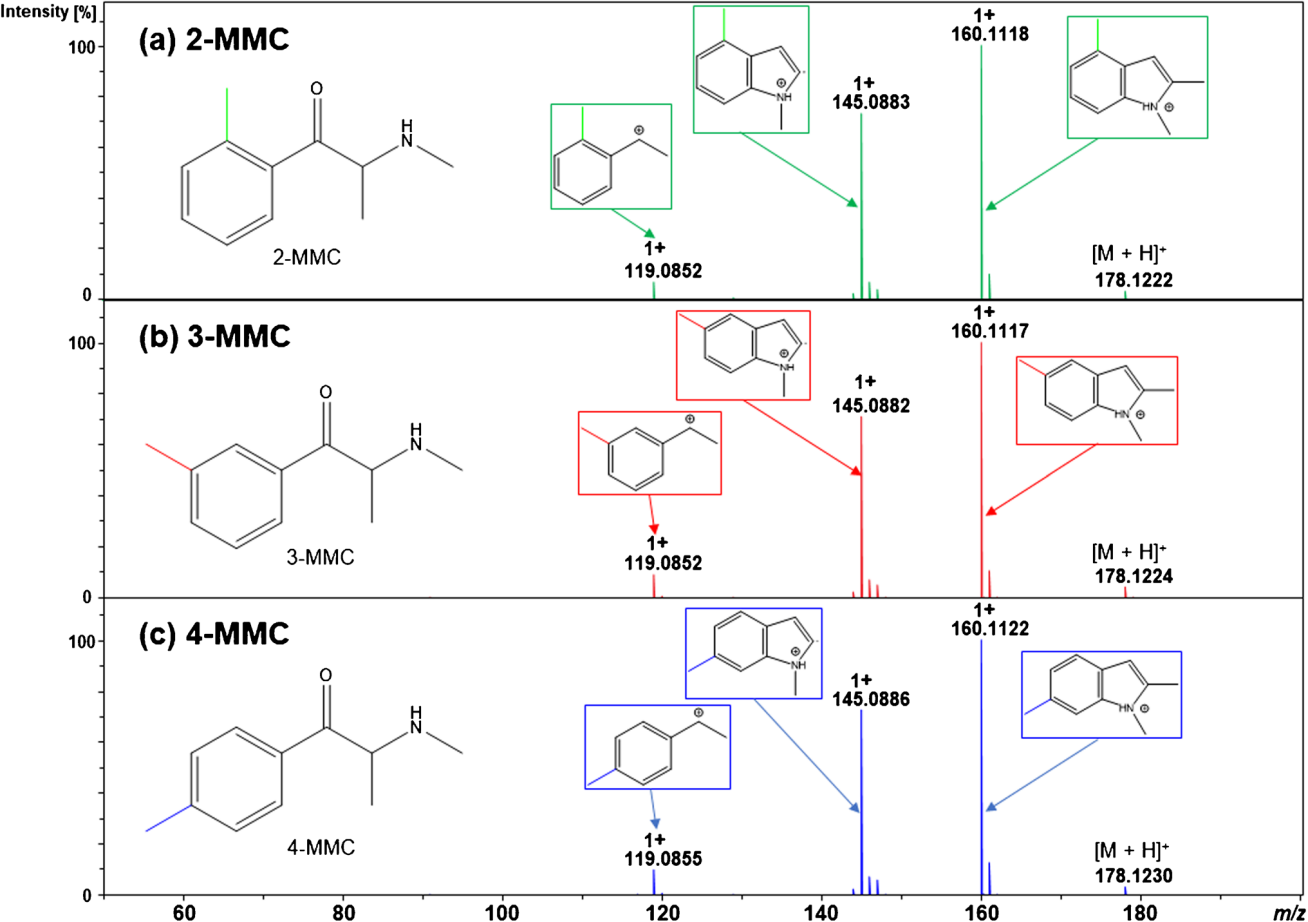
MS/MS spectra observed for 2-MMC (**a**, RT 16.4 min), 3-MMC (**b**, RT 16.5 min), and 4-MMC (**c**, RT 16.5 min) using a collision energy of 17.0 eV and indicating the proposed structures of the obtained product ions. Abbreviations: MMC, methylmethcathinone

MMCs were mostly detected as the [M + H]^+^ protonated precursor (theoretical *m/z* 178.1226). Figure 1 shows the product ion mass spectra observed for 2-MMC, 3-MMC, and 4-MMC.

The product ion mass spectra for MMC isomers revealed abundant *N*-containing product ions, such as the product ions at *m/z* 160.1115 (C_11_H_14_N^+^) and *m/z* 145.0886 (C_10_H_11_N^+^). The dominant loss of H_2_O from the protonated precursor is a common fragmentation behavior for *N*-alkylated synthetic cathinones [39, 40]. In comparison, most other *N*-containing stimulants usually tend to lose the amine group (i.e., Δ = 31 Da) at the beginning of the fragmentation process [20, 21], particularly tertiary amine synthetic cathinones [41]. However, the MMCs favored the loss of H_2_O followed by the loss of a methyl radical (i.e., ^•^CH_3_, Δ = 15 Da). This same fragmentation behavior was observed for most of the metabolites, since they share the same structural backbone. In theory, the loss of radicals from even-electron species is unfavoured under collision-induced dissociation (CID) conditions; however, this phenomenon has been observed for synthetic cathinones under CID conditions by several research groups [20, 39, 42]. For example, Pozo et al. observed a similar fragmentation pattern for 4-MMC and demonstrated that an indole group was formed within 4-MMC due to an in-source intramolecular rearrangement occurring prior to fragmentation in the collision cell [20]. Besides this specific feature, the multiple protonation sites available on MMCs and their metabolites gave rise to diverse and competitive fragmentation patterns, making the interpretation of the MS/MS product ion mass spectra and elucidation of metabolite structures rather challenging, as discussed in the next sections.

### Metabolic profiling of cathinone positional isomers using in vitro metabolic incubations

Since rat liver microsomes and human liver microsomes share similarities in many CYP isoforms, the metabolites of the MMC positional isomers 2-MMC, 3-MMC, and 4-MMC identified in all in vitro metabolic incubations were expected to undergo similar modifications. The metabolism of 4-MMC, for instance, has been well investigated using different in vitro approaches [21, 24]. Most of the phase I metabolites of 4-MMC reported in previous studies were generated by four major metabolic biotransformation routes, namely (i) oxidative *N*-demethylation, (ii) oxidation of the 4-methyl group, (iii) omega-oxidation at the C-3′ position, and (iv) carbonyl reduction, and via a combination of these reactions in the case of secondary metabolites [23]. However, for 2-MMC and 3-MMC, there is no information available in the literature related to their metabolism. The in vitro metabolism of 2-MMC and 3-MMC was therefore investigated.

### Identification of phase I metabolites of 2-methylmethcathinone

For each MMC positional isomer studied (i.e., 2-MMC, 3-MMC and 4-MMC), the fragmentation pattern was first investigated. This information subsequently enabled the tentative structural elucidation of their phase I metabolites. The phase I metabolites of 2-MMC, 3-MMC and 4-MMC identified are listed in Table 1.

**Table 1:** Phase I metabolites of MMC positional isomers detected in liver microsomal incubations using LC-MS/MS

MMC positional isomers	Metabolites	RT (min)	In vitro models	Accurate *m/z* (measured)	Exact mass [M + H]^+^	Chemical formula	Modification(s)	Major product ions (abundance, %)	Error (ppm)*
2-MMC	M1	21.1	pMRLM	178.0878	178.0868	C_10_H_12_NO_2_	Aliphatic hydroxylation or *N*-oxidation; *N*-demethylation; dehydrogenation	160 (100%), 86 (55%), 119 (57%)	5.6
	M2	11.5	pMRLM; pFRLM	194.1191	194.1181	C_11_H_16_NO_2_	Aromatic hydroxylation	176 (100%), 119 (60%), 161 (30%)	5.2
	M3	14.3	pMRLM; pFRLM	194.1191	194.1181	C_11_H_16_NO_2_	Aromatic hydroxylation	176 (100%), 119 (92%), 145 (26%)	5.2
	M4	14.8	pMRLM; pFRLM	194.1191	194.1181	C_11_H_16_NO_2_	Aromatic hydroxylation	176 (100%), 161 (60%), 119 (11%)	5.2
	M5	17.4	pMRLM; pFRLM; pMHLM; pMHLM	194.1189	194.1181	C_11_H_16_NO_2_	Aliphatic hydroxylation or *N*-oxidation	119 (100%), 176 (4%), 74 (2%)	4.1
	M6	16.2	pMRLM; pFRLM; pMHLM; pMHLM	164.1085	164.1075	C_10_H_14_NO	*N*-demethylation	146 (100%), 131 (62%), 119 (14%)	6.1
3-MMC	M7	21.4	pMRLM	178.0862	178.0868	C_10_H_12_NO_2_	Aliphatic hydroxylation or *N*-oxidation; *N*-demethylation; dehydrogenation	160 (100%), 145 (72%), 86 (56%)	-3.4
	M8	8.7	pMRLM; pFRLM; pMHLM; pMHLM	194.1175	194.1181	C_11_H_16_NO_2_	Aliphatic hydroxylation	146 (100%), 158 (64%), 131 (10%)	-3.1
	M9	15.2	pMRLM; pFRLM;	194.1175	194.1181	C_11_H_16_NO_2_	Aromatic hydroxylation	176 (100%), 134 (86%), 161 (64%)	-3.1
	M10	17.6	pMRLM; pFRLM; pMHLM; pMHLM	194.1176	194.1181	C_11_H_16_NO_2_	Aliphatic hydroxylation or *N*-oxidation	119 (100%), 161 (8%), 91 (5%)	-2.6
	M11	15.6	pMRLM; pFRLM; pMHLM; pMHLM	164.1070	164.1075	C_10_H_14_NO	*N*-Demethylation	146 (100%), 131 (58%), 119 (14%)	-3.0
	M12	15.0	pMRLM; pFRLM	210.1124	210.1130	C_11_H_16_NO_3_	Di-hydroxylation or hydroxylation with *N*-oxidation	135 (100%), 174 (5%), 161 (5%)	-2.9
	M 13	6.8	pMRLM; pFRLM	180.1019	180.1025	C_10_H_14_NO_2_	Aliphatic hydroxylation; *N*-demethylation	160 (100%), 145 (78%), 144 (76%)	-3.3
	M 14	19.2	pMRLM	180.1018	180.1025	C_10_H_14_NO_2_	Aliphatic hydroxylation; *N*-demethylation	144 (100%), 162 (73%), 120 (61%)	-3.9
	M15	18.9	pMRLM; pFRLM	192.1021	192.1025	C_11_H_14_NO_2_	Aliphatic hydroxylation; dehydrogenation	146 (100%), 131 (20%), 119 (15%)	-2.1
	M16	16.0	pMRLM	166.1226	166.1231	C_10_H_16_NO	*N*-Demethylation; reduction	148 (100%), 131 (44%), 120 (13%)	-3.0
	M17	11.9	pMRLM; pFRLM	208.0968	208.0974	C_11_H_14_NO_3_	Carboxylation	146 (100%), 85 (64%), 172 (38%)	-2.9
4-MMC	M18	15.7	pMRLM	178.0862	178.0868	C_10_H_12_NO_2_	Aliphatic hydroxylation; *N*-demethylation; dehydrogenation	160 (100%), 145 (72%), 148 (14%)	-3.4
	M19	21.3	pMRLM	178.0866	178.0868	C_10_H_12_NO_2_	Aliphatic hydroxylation or *N*-oxidation; *N*-demethylation; dehydrogenation	160 (100%), 145 (61%), 86 (33%)	-1.1
	M20	6.5	pMRLM; pFRLM; pMHLM; pMHLM	194.1179	194.1181	C_11_H_16_NO_2_	Aliphatic hydroxylation	146 (100%), 158 (70%), 131 (10%)	-1.0
	M21	17.5	pMRLM; pFRLM; pMHLM; pMHLM	194.1180	194.1181	C_11_H_16_NO_2_	Aliphatic hydroxylation or *N*-oxidation	119 (100%), 161 (7%), 91 (4%)	-0.5
	M22	16.3	pMRLM; pFRLM; pMHLM; pMHLM	164.1073	164.1075	C_10_H_14_NO	*N*-Demethylation	146 (100%), 131 (51%), 119 (18%)	-1.2
	M23	14.5	pMRLM; pFRLM	210.1130	210.1130	C_11_H_16_NO_3_	Di-hydroxylation or hydroxylation with *N*-oxidation	135 (100%), 161 (6%), 174 (4%)	0.0
	M24	19.3	pMRLM	180.1025	180.1025	C_10_H_14_NO_2_	Aliphatic hydroxylation; *N*-demethylation	160 (100%), 145 (91%), 144 (44%)	0.0
	M25	16.1	pMRLM	166.1230	166.1231	C_10_H_16_NO	*N*-Demethylation; reduction	148 (100%), 131 (44%), 120 (13%)	-0.6

*The error refers to the relative mass difference expressed in ppm between the measured accurate mass of the metabolites and their theoretical exact mass. Abbreviations: *MMC*, methylmethcathinone; *RT*, retention time; *pMRLM*, pooled male rat liver microsomes; *pFRLM*, pooled female rat liver microsomes; *pMHLM*, pooled male human liver microsomes; *pFHLM*, pooled female human liver microsomes

#### M1

This metabolite was only detected in pMRLM incubations, leading to a protonated precursor ion at *m/z* 178.0878 (C_10_H_12_NO_2_^+^). The empirical formula of the protonated precursor ion was derived based on the accurate mass measurement (see Table 1). The **M1** metabolite was formed from a combination of three metabolic reactions, namely, *N*-demethylation, *N*-oxidation, or omega-oxidation at the C-3′ position, followed by dehydrogenation. The abundant diagnostic iminium ions at *m/z* 86.0235 (C_3_H_4_NO_2_^+^) and *m/*z 58.0294 (C_2_H_4_NO^+^) indicate that an *N*-demethylation occurred and a carbonyl group was introduced to the aliphatic chain. Yet, the MS/MS product ion mass spectrum of this metabolite is not sufficient to inform about the exact location where the carbonyl was introduced in the aliphatic chain, which needs further investigation. Additional ions that support this structural modification include product ions at *m/z* 142.0649 (C_10_H_8_N^+^), *m/z* 119.0489 (C_8_H_7_O^+^), and *m/z* 91.0540 (C_7_H_7_^+^). The product ion mass spectra of many synthetic cathinones contain a tropylium ion at nominal *m/z* 91, particularly for synthetic cathinones with at least four carbon alkyl chains [43], although in this case the presence of the methyl substitution to the aromatic ring leads to the formation of the methyl-substituted benzoylium ion (i.e., *m/z* 119.0489) and the tropylium ion through the loss of CO (i.e., Δ = 28 Da). Interestingly, some artifact ions were also observed in the product ion mass spectrum, such as the abundant ions at *m/z* 160.1118 (C_11_H_14_N^+^) and *m/z* 145.0890 (C_10_H_11_N^+^), which could not have formed from **M1** given their elemental compositions and the accurate mass measurements acquired with the high-resolution mass analyzer (TOF/MS) instrument used in this study. Strikingly, the results obtained with UHPLC-MS/MS analysis revealed a lack of product ions at *m/z* 160.1118 (C_11_H_14_N^+^) and *m/z* 145.0890 (C_10_H_11_N^+^), which suggests that an interference co-eluted with **M1** under conventional LC separation conditions but was resolved using UHPLC conditions.

#### M2–5

These four metabolites were all detected in pooled female and male rat liver microsomal (pRLM) incubations, but **M2**–**M4** were not found in female and male human liver microsomal (pHLM) incubations, with a protonated precursor ion at *m/z* 194.1170 (C_11_H_16_NO_2_^+^). This accurate mass measurement indicates that these metabolites show one additional oxygen atom compared to the parent compound 2-MMC (Δ = 16 Da). Since 2-MMC contains multiple potential hydroxylation sites, their respective MS/MS spectra were investigated to propose the most feasible oxidation site for each metabolite. **M2**, **M3**, and **M4** all generated the diagnostic product ion at nominal *m/z* 135 corresponding to the hydroxylated methylbenzoylium ion (C_8_H_7_O_2_^+^). In addition, only a single loss of H_2_O was observed from their respective MS/MS product ion mass spectra, which also supports that one oxygen atom was introduced to an undetermined aromatic position. Additional support for the presence of hydroxylation on the aromatic ring includes the presence of product ions at nominal *m/z* 161, *m/z* 145, and *m/z* 119. The product ion at nominal *m/z* 161 (C_10_H_11_NO^+^) is formed through the loss of a methyl radical from the primary product ion at nominal *m/z* 176 (C_11_H_14_NO^+^), which is formed through the loss of H_2_O from the protonated precursor ion for **M2**–**M4**. The methyl radical (i.e., ^•^CH_3_) can be lost from either the amine or aliphatic chain [20], which contributes to the abundance of this fragmentation pathway. The loss of the amine moiety from the primary product ion at nominal *m/z* 176 leads to the formation of the product ion at nominal *m/z* 145 (C_10_H_9_O^+^). Subsequent loss of ethyne (i.e., C_2_H_2_) results in the formation of the product ion at nominal *m/z* 119 (C_8_H_7_O^+^), still containing the hydroxylated substitution. **M5** was also detected in all metabolic incubations. From the MS/MS product ion mass spectrum of **M5**, the presence of the methyl-substituted benzoylium ion at *m/z* 119.0502 (C_8_H_7_O^+^) supports that the hydroxylation cannot have occurred on the aromatic ring. A typical single loss of H_2_O with a nominal mass shift of 18 Da and a double loss of H_2_O with a nominal mass shift of 36 Da were observed. Yet, the MS/MS product ion mass spectrum of **M5** is not sufficient to reveal the exact location where oxygen was exactly introduced in the aliphatic chain, which also needs further investigation. Pedersen et al. report a similar issue when elucidating the structure of one hydroxylated metabolite of methylone (i.e., methylenedioxymethcathinone), whose CID product ion spectrum was dominated by only one abundant ion at *m/z* 149.0243 (C_8_H_5_O_3_^+^). Therefore, they proposed two possible structures for that metabolite [44], a strategy we have also opted for in the present study.

#### M6

The empirical formula of this metabolite (C_10_H_14_NO^+^) suggests that it originates from *N*-demethylation (Δ =  − 14 Da). The product ion mass spectrum followed a similar fragmentation behavior as observed for the parent 2-MMC, including the dominant loss of H_2_O followed by the loss of a methyl radical to form product ions at *m/z* 146.0978 (C_10_H_12_N^+^) and *m/z* 131.0742 (C_9_H_9_N^•+^), respectively. This metabolite was detected in each of the pRLM and pHLM incubations as the most abundant metabolite observed for 2-MMC.

**Fig. 2:**
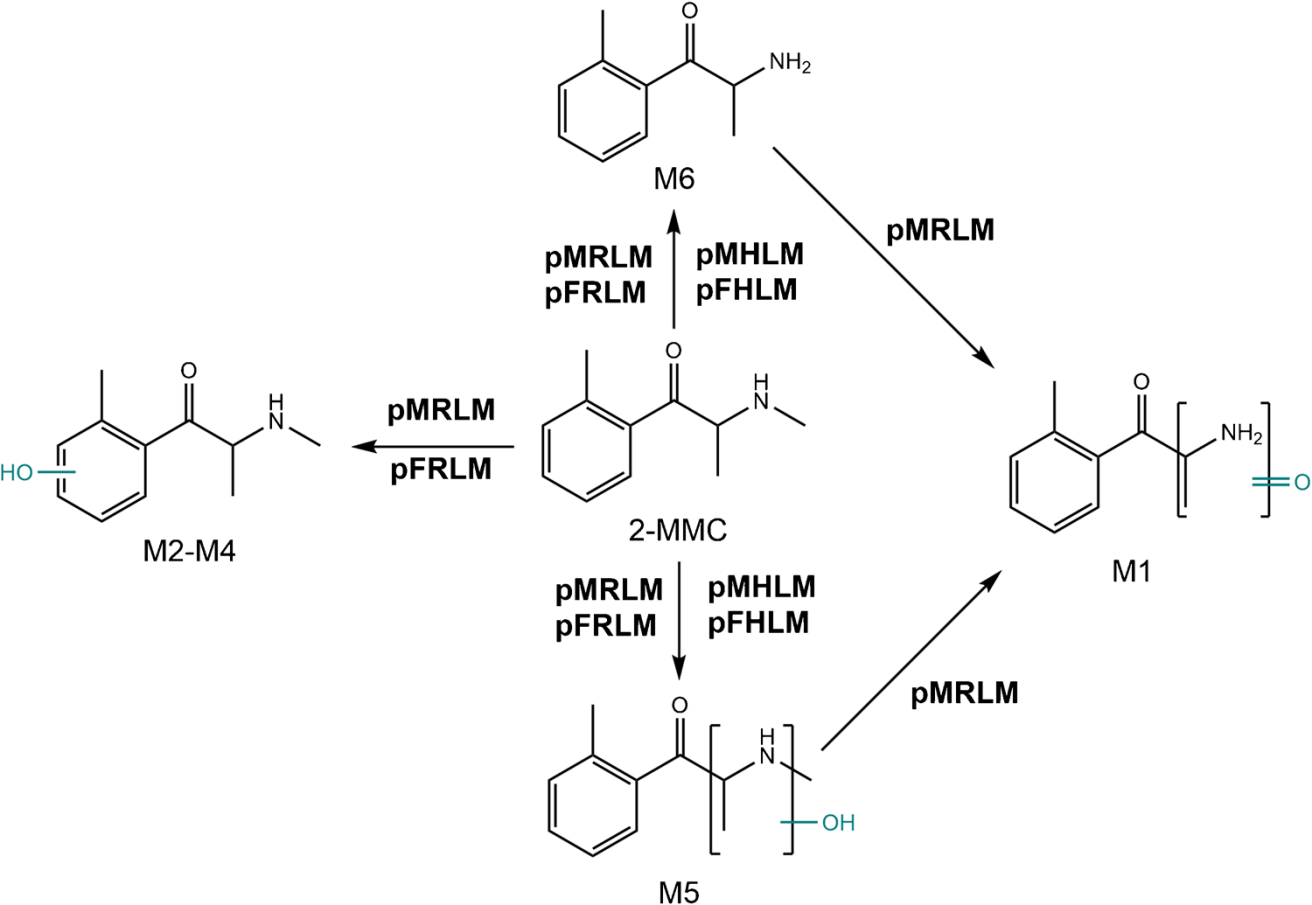
Proposed metabolic pathways of 2-MMC obtained with liver microsomal incubations. The metabolite numbers correspond to the identified metabolites listed in Table 1. Abbreviations: MMC, methylmethcathinone; pMRLM, pooled male rat liver microsomes; pFRLM, pooled female rat liver microsomes; pMHLM, pooled male human liver microsomes; pFHLM, pooled female human liver microsomes

The in vitro phase I metabolic biotransformation of 2-MMC using pooled male and female rat and human liver microsomal incubations is proposed in Fig. 2.

### Identification of phase I metabolites of 3-methylmethcathinone

#### M7

This metabolite was only detected in pMRLM incubations, with a protonated precursor ion detected at *m/z* 178.0862 (C_10_H_12_NO_2_^+^). **M7** and **M1** share the same modifications through a combination of three metabolic reactions, i.e., *N*-demethylation, *N*-oxidation, or omega-oxidation at the C-3′ position, followed by dehydrogenation. The MS/MS product ion mass spectrum of this metabolite (see Fig. S1) was similar to the MS/MS product ion mass spectrum of **M1**, including the presence of diagnostic iminium ions detected at *m/z* 86.0237 (C_3_H_4_NO_2_^+^) and *m/z* 58.0301 (C_2_H_4_NO^+^) and supporting information about the location of substitution through product ions at *m/z* 142.0649 (C_10_H_8_N^+^), *m/z* 119.0490 (C_8_H_7_O^+^), and *m/z* 91.0540 (C_7_H_7_^+^). The structure of **M7** was therefore elucidated following the same procedure as **M1**, based on the presence of the same product ions in both MS/MS product ion mass spectra. Similar to **M1**, there was evidence of co-elution due to the presence of product ions at *m/z* 160.1120 (C_11_H_14_N^+^) and *m/z* 145.0886 (C_10_H_11_N^+^), which could not have been formed from **M7** given the accurate mass measurements. The results from the UHPLC-MS/MS analysis revealed a lack of product ions at *m/z* 160.1120 (C_11_H_14_N^+^) and *m/z* 145.0886 (C_10_H_11_N^+^), which suggests that the interference co-eluted with **M7** under conventional LC separation conditions, but was resolved under UHPLC conditions. The extracted ion chromatogram (EIC) and MS/MS product ion mass spectrum of the metabolite **M7**, including the proposed fragmentation patterns, are included in Fig. S1.

#### M8–10

These three metabolites were all detected in pRLM incubations, showing a protonated precursor ion at *m/z* 194.1175 (C_11_H_16_NO_2_^+^). Yet, only **M8** and **M10** were discovered from pHLM incubations. The measured accurate mass at *m/z* 194.1175 suggests that these metabolites contain one more oxygen atom than 3-MMC (Δ = 16 Da). Since 3-MMC contains several potential hydroxylation sites, the respective MS/MS product ion mass spectra were used to propose the most likely structure for each metabolite. The MS/MS product ion mass spectrum of **M8** showed a primary H_2_O loss at *m/z* 176.1068 (C_11_H_14_NO^+^), followed by either a second H_2_O loss to obtain a product ion detected at *m/z* 158.0963 (C_11_H_12_N^+^) or the loss of formaldehyde (CH_2_O) to obtain a product ion at *m/z* 146.0963 (C_10_H_12_N^+^). The structure of **M8** was therefore proposed to be 3-hydroxymethylmethcathinone (3-OH-MMC), which was also found in biosamples of 3-MMC-related cases previously reported [45, 46]. It is worth mentioning that such a hydroxylated metabolite was not detected for 2-MMC. For the less abundant **M9**, the diagnostic ion at *m/z* 135.0435 (C_8_H_7_O_2_^+^) and the single loss of H_2_O both indicate that one oxygen atom was likely introduced to the aromatic ring. However, a product ion at *m/z* 134.0963 (C_9_H_12_N^+^) was also detected, but its tentative structure could not be determined confidently. With UHPLC-MS/MS analysis, the resulting product ion mass spectrum of **M9** did not show any product ion at *m/z* 134.0963, but an additional diagnostic product ion at *m/z* 107.0499 (C_7_H_7_O^+^) was detected, which provided further support for the elucidation that the hydroxyl group was introduced to the aromatic ring (Fig. S2. f). **M10** was formed following a similar transformation as **M5**, i.e., with the introduction of a hydroxyl group to the aliphatic chain. Two sequential losses of H_2_O were observed leading to product ions detected at *m/z* 176.1085 (C_11_H_14_NO^+^) and 158.0969 (C_11_H_12_N^+^), suggesting the presence of a hydroxyl group in the aliphatic chain. However, the MS/MS product ion mass spectrum of **M10** is again not sufficient to determine the exact location where the oxygen was introduced in the aliphatic chain. The EIC and MS/MS product ion mass spectra of the **M8**–**10** metabolites, including the proposed fragmentation patterns, are shown in Fig. S2.

#### M11

The empirical formula of this metabolite (C_10_H_14_NO^+^) suggests an *N*-demethylated metabolite (Δ =  -14 Da) due to the loss of a methyl group from 3-MMC. This was also consistent with the measured accurate mass at *m/z* 164.1070. This de-alkylated metabolite was also detected in each of the pRLM and pHLM incubations as the most abundant species of 3-MMC. The EIC and MS/MS product ion mass spectrum of **M11**, including the proposed fragmentation pattern, are illustrated in Fig. S3. The sequential loss of H_2_O and a methyl radical led to the formation of product ions at *m/z* 146.0936 (C_10_H_12_N^+^) and *m/z* 131.0728 (C_9_H_9_N^+^), respectively.

#### M12

This metabolite was only detected in pRLM incubations, with a protonated precursor ion detected at *m/z* 210.1124 (C_11_H_16_NO_3_^+^). Based on its accurate mass, this metabolite was produced via addition of two oxygen atoms to 3-MMC (Δ = 32 Da). The product ion mass spectrum showed an abundant ion at *m/z* 135.0435 (C_8_H_7_O_2_^+^), indicating aromatic hydroxylation. There were also less abundant product ions at *m/z* 192.1021 (C_11_H_14_NO_2_^+^) and *m/z* 174.0913 (C_11_H_12_NO^+^) formed through the sequential loss of H_2_O. Secondary fragmentation from the intermediate ion at *m/z* 192.1021 (C_11_H_14_NO_2_^+^) revealed the loss of a methanol radical (^•^CH_2_OH, Δ = 31 Da) to form a product ion at *m/z* 161.0835 (C_10_H_11_NO^+^) that provided further confirmation of oxidation on the aliphatic chain, *N*-oxidation, or omega-oxidation in the C-3′ position. Figure S4 shows the EIC and MS/MS product ion mass spectrum of **M12**, as well as the proposed fragmentation pattern.

#### M13 and M14

These two metabolites were only detected in pRLM incubation, leading to a protonated precursor ion at *m/z* 180.1018 (C_10_H_14_NO_2_^+^), and the accurate mass measurements identified these metabolites as compounds with hydroxylation and de-methylation. However, **M14** was generated exclusively in pMRLM incubations. The product ion mass spectrum of **M13** showed that there was a primary H_2_O loss to form the product ion at *m/z* 162.0911 (C_10_H_12_NO^+^), followed by either a second H_2_O loss to *m/z* 144.0807 (C_10_H_10_N^+^) or the loss of formaldehyde (CH_2_O) to *m/z* 132.0807 (C_9_H_10_N^+^), yet the product ions at *m/z* 160.1115 (C_11_H_14_N^+^) and *m/z* 145.0882 (C_10_H_11_N^+^) could not have originated from **M13** and, thus, must have originated by another metabolite that co-eluted and was present within the 4 Da isolation window. Nonetheless, such contradictory product ions were not detected using UHPLC-MS/MS. The structure of this metabolite was tentatively elucidated as 3-hydroxymethyl-*N*-demethyl-MMC. **M14** showed that there was a primary H_2_O loss to form the product ion at *m/z* 162.0913 (C_10_H_12_NO^+^), followed by either a second H_2_O loss to *m/z* 144.0808 (C_10_H_10_N^+^) or the loss of CO to form *m/z* 134.0966 (C_9_H_12_N^+^), indicating the likely inclusion of a hydroxyl group to the C-3′ position. The EIC and MS/MS product ion mass spectra of 3-MMC **M13** and **M14**, including the proposed fragmentation patterns, are illustrated in Fig. S5.

#### M15

This metabolite was detected in pRLM incubations only. The protonated precursor ion at *m/z* 192.1021 (C_11_H_14_NO_2_^+^) indicates that hydroxylation and dehydrogenation occurred. The sequential loss of CO and H_2_O was observed through the detection of product ions at *m/z* 164.1069 (C_10_H_14_NO^+^) and *m/z* 146.0964 (C_10_H_12_N^+^), respectively. The structure of this metabolite was identified as 3′-aldehyde-MMC. The EIC and MS/MS product ion mass spectrum of **M15**, including the proposed fragmentation pattern, are illustrated in Fig. S6.

#### M16

This metabolite was detected in pMRLM incubations only. The protonated precursor ion at *m/z* 166.1226 (C_10_H_16_NO^+^) indicates that carbonyl reduction and *N*-demethylation occurred; this metabolite was therefore elucidated as 3-methylnorephedrine. The fragmentation of this metabolite is different from the other metabolites in this study, as the reduction of carbonyl inhibited indole ring formation. Such biotransformation was not observed with 2-MMC metabolic incubations. In addition to the lack of indole ring formation, **M16** fragmentation also included the formation of a product ion at *m/z* 131.0855 (C_10_H_11_^+^) formed through the loss of ammonia (NH_3_) as the secondary fragmentation of the primary product ion at *m/z* 148.1121 (C_10_H_14_N^+^) formed through the loss of H_2_O. The EIC and MS/MS product ion mass spectrum of **M16**, including the proposed fragmentation pattern, are illustrated in Fig. S7.

#### M17

This metabolite was only detected in pRLM incubations. The protonated precursor ion at *m/z* 208.0968 (C_11_H_14_NO_3_^+^) indicates that carboxylation most likely occurred. The diagnostic product ions for this elucidation are *m/z* 190.0856 (C_11_H_12_NO_2_^+^) formed through the loss of H_2_O and the subsequent product ion at *m/z* 146.0967 (C_10_H_12_N^+^) formed through the loss of CO_2_, which is characteristic for an aromatic carboxylic acid. This metabolite was therefore elucidated as 4-carboxymethcathinone. The EIC and MS/MS product ion mass spectrum of **M17**, including the proposed fragmentation pattern, are illustrated in Fig. S8.

**Fig. 3:**
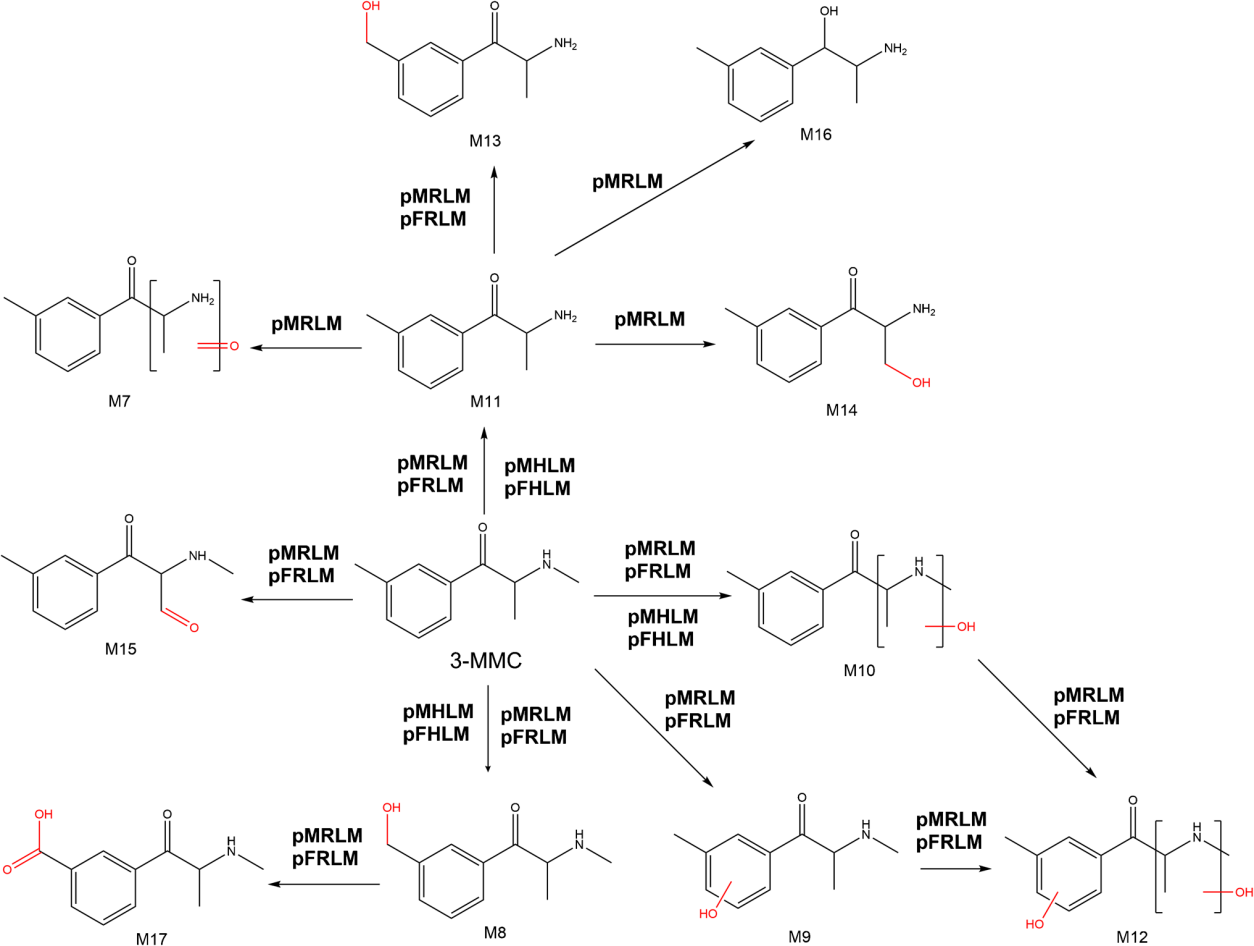
Proposed metabolic pathways of 3-MMC obtained with pooled liver microsomal incubations. The metabolite numbers correspond to the identified metabolites listed in Table 1. Abbreviations: MMC, methylmethcathinone; pMRLM, pooled male rat liver microsomes; pFRLM, pooled female rat liver microsomes; pMHLM, pooled male human liver microsomes; pFHLM, pooled female human liver microsomes

The in vitro phase I metabolites of 3-MMC, for the first time reported, are illustrated in Fig. 3.

### Identification of phase I metabolites of 4-methylmethcathinone

#### M18 and M19

These two metabolites were only detected in pMRLM incubations, leading to a protonated precursor ion at *m/z* 178.0866 (C_10_H_12_NO_2_^+^). **M19** underwent the same biotransformations as **M1** and **M7** from a combination of three metabolic reactions, which were *N*-demethylation, *N*-oxidation or omega-oxidation at the C-3′ position, followed by dehydrogenation. The MS/MS product ion mass spectrum of this metabolite was similar to **M1** and **M7**, including the diagnostic ion at nominal *m/z* 86 (C_3_H_4_NO_2_^+^). This metabolite is reported for the first time. Compared with **M19**, **M18** is more polar, as demonstrated by a H_2_O loss to the product ion at *m/z* 160.0759 (C_10_H_10_NO^+^) and the loss of formaldehyde (CH_2_O) to obtain a product ion at *m/z* 148.0759 (C_9_H_10_NO^+^). Another H_2_O loss was observed from the product ion at *m/z* 148.0759 (C_9_H_10_NO^+^), giving rise to the product ion at *m/z* 130.0654 (C_9_H_8_N^+^). All these fragmentation patterns suggest that the hydroxyl was introduced to the benzyl group. The other modification to **M18** is likely due to the dehydrogenation based on the measured accurate mass of the protonated precursor ion. Nevertheless, some artifact ions were observed in the product ion mass spectrum, such as the product ion at *m/z* 160.1125 (C_11_H_14_N^+^) and the one at *m/z* 145.0890 (C_10_H_11_N^+^), which could not have originated from **M18**. Such contradictory product ions are likely due to co-elution, as they were not observed in the UHPLC-MS/MS product ion mass spectrum for **M18**.

#### M20 and M21

These two metabolites were detected in pRLM and pHLM incubations, leading to a protonated precursor ion at *m/z* 194.1179 (C_11_H_16_NO_2_^+^); the accurate mass measurements indicated the presence of hydroxylation (Δ = 16 Da). **M20** had a similar fragmentation pattern as **M8**, which helped with the structural elucidation of **M20** as 4-hydroxymethylmethcathinone (4-OH-MMC). This metabolite is a primary phase I metabolite which has been identified by previous studies [18–22]. The MS/MS product ion mass spectrum of **M21** was dominated by the product ion at *m/z* 119.0487 (C_8_H_7_O^+^), indicating that the aromatic moiety of 4-MMC remains intact. Previous study detected this metabolite in rat blood after oral administration and speculated that the hydroxylation was introduced to the C-3′ position based on the observation of two H_2_O losses and the product ion at *m/z* 74.0591 (C_3_H_8_NO^+^) [22]. However, the MS/MS product ion mass spectrum of **M21** is not sufficient to determine the exact location where the oxygen atom was introduced to the aliphatic chain. A previous study also discovered an aromatic monohydroxylated metabolite from 4-MMC in vivo [22], yet this metabolite was not detected here.

#### M22

The empirical formula of this metabolite (C_10_H_14_NO^+^) suggests the *N*-demethylated metabolite (Δ = -14 Da). This is also consistent with the accurate mass measurement at *m/z* 164.1073. This de-methylated metabolite was also the most abundant species of 4-MMC and was found in all in vitro metabolic incubations in this study and had been reported by previous research in the metabolism of 4-MMC [19–23].

#### M23

The empirical formula of this metabolite (C_11_H_16_NO_3_^+^) suggests a di-hydroxylation metabolite with two diagnostic ions, namely the product ion at *m/z* 161.0828 (C_10_H_11_NO^+^) and the product ion at *m/z* 135.0437 (C_8_H_7_O_2_^+^), indicating one hydroxylation is located at the aromatic ring. This metabolite was only detected in pRLM incubations. The MS/MS product ion mass spectrum of this metabolite was similar to the MS/MS product ion mass spectrum of **M12**. The structure of **M23** was therefore elucidated following the same procedure as** M12.**

#### M24

This metabolite was exclusively detected in pMRLM incubations, leading to a protonated precursor ion at *m/z* 180.1025 (C_10_H_14_NO_2_^+^), and the accurate mass measurement indicates this metabolite as a compound with hydroxylation and *N*-demethylation. The product ion mass spectrum showed the sequential loss of H_2_O to the product ions at *m/z* 162.0916 (C_10_H_12_NO^+^) and *m/z* 144.0811 (C_10_H_10_N^+^), as observed previously for **M14**. The combination of these observations and the relatively longer retention time suggests that the hydroxylation probably occurred at the C-3′ position.

#### M25

This metabolite was only detected in pMRLM incubations. The protonated precursor ion at *m/z* 166.1230 (C_10_H_16_NO^+^) indicates that reduction of the carbonyl group and *N*-demethylation occurred. The fragmentation pattern of this metabolite is the same as **M16**; therefore, **M25** was identified as 4-methylnorephedrine. This metabolite was also discovered by previous studies [20, 23, 24]. However, such biotransformation was only observed in pMRLM incubations of 4-MMC.

**Fig. 4:**
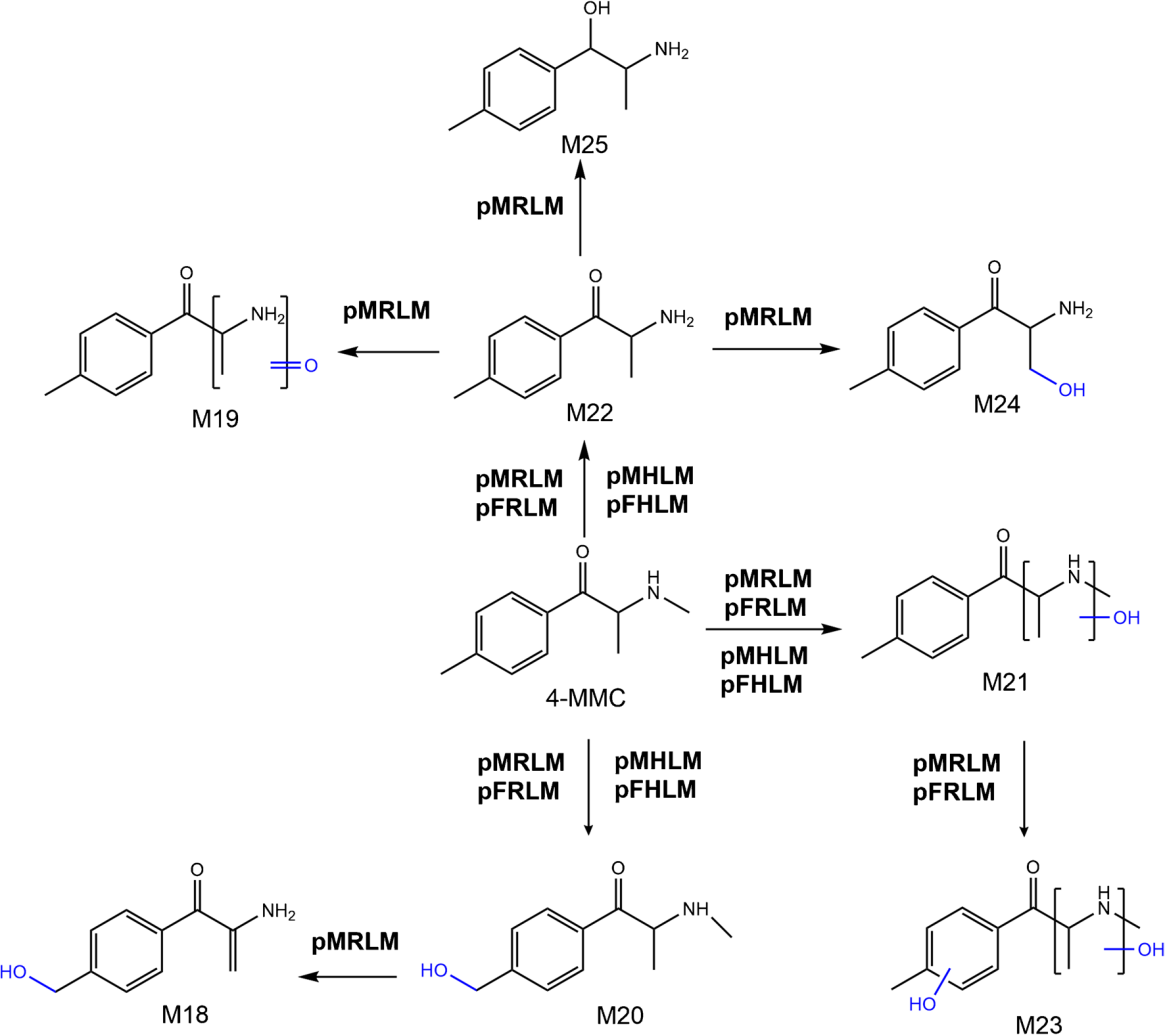
Proposed metabolic pathways of 4-MMC obtained with liver microsomal incubations. The metabolite numbers correspond to the identified metabolites listed in Table 1. Abbreviations: MMC, methylmethcathinone; pMRLM, pooled male rat liver microsomes; pFRLM, pooled female rat liver microsomes; pMHLM, pooled male human liver microsomes; pFHLM, pooled female human liver microsomes

The metabolic pathway of 4-MMC is proposed in Fig. 4. Some phase I metabolites of 4-MMC deciphered in this study, such as **M20**, **M22**, and **M25**, are in accordance with metabolites reported in previous studies [20–24].

Overall, a total of 25 phase I metabolites of MMC positional isomers were detected and identified using pHLM and pRLM incubations. The accurate mass measurement for each metabolite enabled the determination of only one feasible elemental formula at the selected mass window (± 5 mDa). Among the metabolites detected, six phase I metabolites were detected from 2-MMC, eleven phase I metabolites from 3-MMC*,* and eight phase I metabolites from 4-MMC in vitro metabolic models, respectively. Some phase I metabolites from the MMC positional isomers were also positional isomers to each other, such as **M8** and **M20**, thus sharing very similar fragmentation patterns with each other as their parent compounds do*.* Notably, for some metabolites, such as **M1**, **M5**, **M7**, **M10**, **M12**, **M19**, **M21**, and **M23**, two possible structures were proposed for each, as their MS/MS product ion mass spectra were not sufficient to confidently determine a unique structure. Since the in vitro metabolism of 3-MMC using pRLM incubations is the most extensive of these metabolic incubations, the fragmentation patterns of 3-MMC metabolites are detailed in Supplementary Fig. S1–S8. A summary of all the phase I metabolites of the MMC positional isomers formed using liver microsomal incubations is shown in Table 1. Seventeen phase I metabolites were detected in pRLM incubations only, and not in pHLM incubations. Furthermore, eight metabolites were detected in both pRLM and pHLM incubations. There were a total of 25 metabolites detected in pRLM incubations; among those, seven metabolites were observed with pMRLM incubations only but not in pFRLM incubations.

### Phase I metabolism of methylmethcathinone positional isomers: general trends

**Fig. 5:**
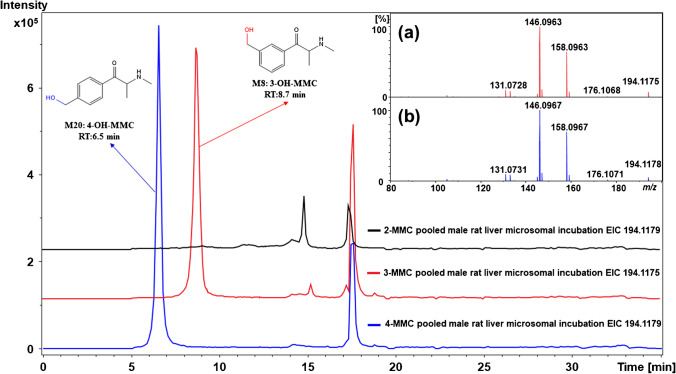
Extracted ion chromatograms (EICs) obtained for monohydroxylated metabolites in pooled male rat liver microsomal incubations. (**a**) Product ion mass spectrum observed for 3-OH-MMC. (**b**) Product ion mass spectrum observed for 4-OH-MMC. The metabolite numbers correspond to the identified metabolites listed in Table 1. The retention times observed for 3-OH-MMC (8.7 min) and 4-OH-MMC (6.5 min) were significantly different. No hydroxylated metabolite was observed for 2-MMC

*N*-Demethylation occurred for the three MMC positional isomers via the enzymatic removal of a methyl group from the nitrogen atom, resulting in the most abundant metabolites detected, namely, **M6 (**from 2-MMC), **M11** (from 3-MMC), and **M22** (from 4-MMC). Also, different hydroxylated metabolites were observed in pHLM and pRLM incubations for the MMC positional isomers. For instance, the oxidation of the methyl group at the aromatic ring was observed for both 3-MMC and 4-MMC, resulting in the formation of **M8** and **M20**, respectively, but not for 2-MMC with its methyl group in the *ortho*-position, as illustrated in Fig. 5. These two metabolites, 3-OH-MMC (**M8**) and 4-OH-MMC (**M20**), are interesting as they can be used as specific biomarkers for discriminating between the consumption of 3-MMC and 4-MMC. Indeed, the metabolites **M8** and **M20** eluted at 6.5 min and 8.7 min for 4-OH-MMC and 3-OH-MMC, respectively, showing a sufficient resolution to enable a clear discrimination, while their parent compounds 3-MMC and 4-MMC are often difficult to separate and distinguish from each other using conventional analytical methods [16, 47]. The analysis of such hydroxylated metabolites in body fluids may help with the identification of 3-MMC consumption and avoid the possible underestimation of the real number of cases of 3-MMC use and, in turn, its toxicity. In addition to the difference in the formation of monohydroxylated metabolites, the dehydrogenated metabolites were also only detected for 3-MMC and 4-MMC, namely, **M16** and **M25**.

Besides the difference in aliphatic hydroxylated metabolites observed in this study, aromatic hydroxylation metabolites were only detected for 2-MMC and 3-MMC. Additionally, di-hydroxylated metabolites (**M12** and **M23**) were observed for the metabolism of 3-MMC and 4-MMC, but not for 2-MMC.

### Differences in biotransformation of methylmethcathinone positional isomers in pooled rat liver microsomes vs. pooled human liver microsomes

**Fig. 6:**
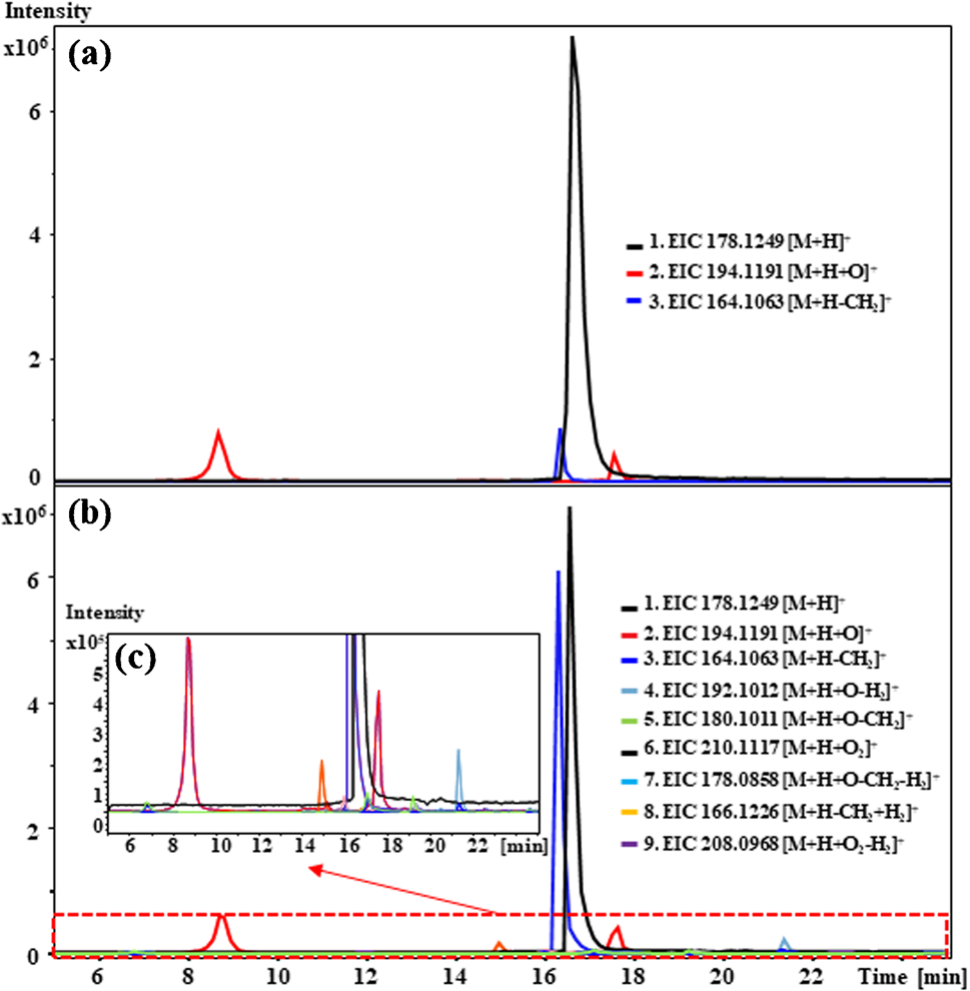
Comparisons of the extracted ion chromatograms (EICs) obtained for the metabolites of 3-MMC in pooled male human liver microsomal (pMHLM) and pooled male rat liver microsomal (pMRLM) incubations. (**a**) EICs of 3-MMC, **M8**, **M10**, and **M11**, which were observed in pMHLM incubations. (**b**) EICs of 3-MMC and **M7**–**M17**, which were generated from pMRLM incubations. (**c**) EICs of the less abundant metabolites generated from pMRLM incubations

The biotransformation of MMC positional isomers showed significant differences in pHLM and pRLM incubations, where more metabolites were detected with pRLM incubations compared to pHLM incubations, as illustrated in Fig. 6. All the metabolites which were detected in pHLM incubations, i.e., **M5**, **M6**, **M8**, **M10**, **M11**, **M17**, **M18**, and **M19**, were the same as those observed with pRLM incubations (Table 1). These metabolites were derived from oxidative biotransformation via either hydroxylation or *N*-demethylation, resulting in an array of primary phase I metabolites without secondary modifications. Strikingly, secondary metabolites, such as **M12** and **M13**, arising from combinations of microsomal oxidation reactions, were observed with pRLM incubations only (Fig. 6b). Moreover, metabolites arising from aromatic hydroxylation were only detected in pRLM incubations.

Previous in vitro studies have demonstrated that CYP2D6 is the main enzyme responsible for the metabolism of 4-MMC, along with minor contributions from other drug-metabolizing enzymes, such as CYP1A2, CYP2C19, CYP2C9, and CYP3A4 [21]. The pHLM material used contained a pool of microsomes all derived from Caucasians (*n* = 10 for each sex), which may explain why fewer metabolites were detected, as European Caucasians show a much lower level of CYP2D6 activity compared with other ethnicities due to genetic polymorphism [48]. About 7% of Caucasians are devoid of CYP2D6 activity, as they show two inactive CYP2D6 alleles and, thus, do not synthesize the active enzyme. The expression of cytochrome P450s and other drug-metabolizing enzymes in human is probably not influenced by sex as much as in rats [49]. The pRLM in vitro metabolic incubations of each sex have sex-specific enzymes (i.e., CYP2A2, CYP2C11, CYP2C13, and CYP3A2) which are male-specific enzymes in sexually mature male rats, and CYP2C12 is a sex-specific enzyme in female rats, which consequently may have led to the formation of sex-specific metabolites.

### Sex-specific metabolism of methylmethcathinone positional isomers

**Fig. 7:**
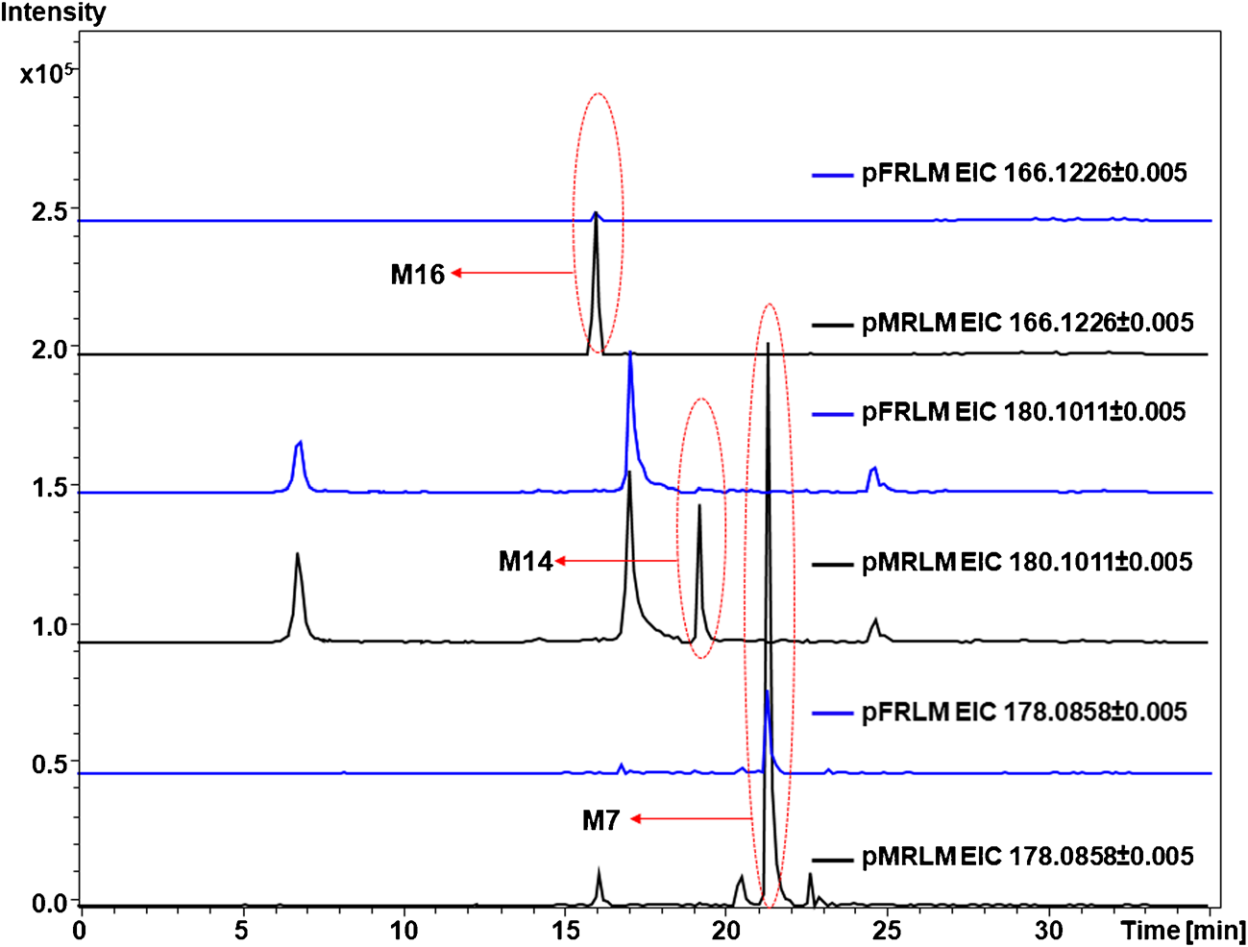
Comparisons of extracted ion chromatograms (EICs) of the discriminative metabolites **M7**, **M14**, and **M16** of 3-MMC measured in pooled male rat liver microsomal (pMRLM) incubations (black EIC traces) which were not detected in pooled female rat liver microsomal (pFRLM) incubations (blue EIC traces). The metabolite numbers correspond to the identified metabolites listed in Table 1

The analysis of the possible sex-specific differences in the metabolism of MMC positional isomers using liver microsomal incubations showed relevant differences. Notably, more metabolites were observed in pMRLM compared with pFRLM incubations, as illustrated in Fig. 7. Indeed, seven metabolites of MMCs, all secondary metabolites (Table 1), were exclusively detected in pMRLM incubations, namely, **M1**, **M7**, **M14**, **M16**, and **M21**. The metabolites **M1**, **M7**, and **M16** underwent the same modifications as their respective parent compound, namely, *N*-demethylation and hydroxylation followed by dehydrogenation. In addition, the metabolites **M14** and **M21** were also further metabolized via the same pathway, i.e., *N*-demethylation and hydroxylation. The EICs of **M7** and **M14** observed for both sexes are shown in Fig. 6. Supplementary Fig. S9 shows the EICs of additional metabolites that were different between pMRLM and pFRLM incubations.

Sex-specific differences in oxidative metabolism of some illicit drugs, such as Δ^9^-tetrahydrocannabinol (Δ^9^-THC) from marijuana [36], MDMA [50], and ( +)-methamphetamine [51], were previously reported as well. The sex differences in metabolism of Δ^9^-THC were attributed to the different forms of P450s in liver microsomes of male and female rats. Δ^9^-THC in female rats is metabolized into the active and potent metabolite 11-hydroxy-Δ^9^-THC dominantly by CYP2C6, yet male rats tend to bio-transform Δ^9^-THC into multiple metabolites via CYP2C11 rather than 11-hydroxy-Δ^9^-THC [36]. In vitro study of MDMA and methylenedioxyamphetamine (MDA) metabolism showed that MDMA was biotransformed into MDA more rapidly in male rat liver microsomes than female rat liver microsomes [49]. Additionally, sex- and dose-dependent differences in ( +)-methamphetamine pharmacokinetics and metabolism were also observed in male and female Sprague-Dawley rats [51]. Sex-specific differences in metabolism of xenobiotics are also of significant importance from a toxicological standpoint, as the difference may produce a lower clearance of the compound, thus leading to a prolonged half-life, higher blood concentration of the parent compound, and, in turn, increased toxicity.

**Fig. 8:**
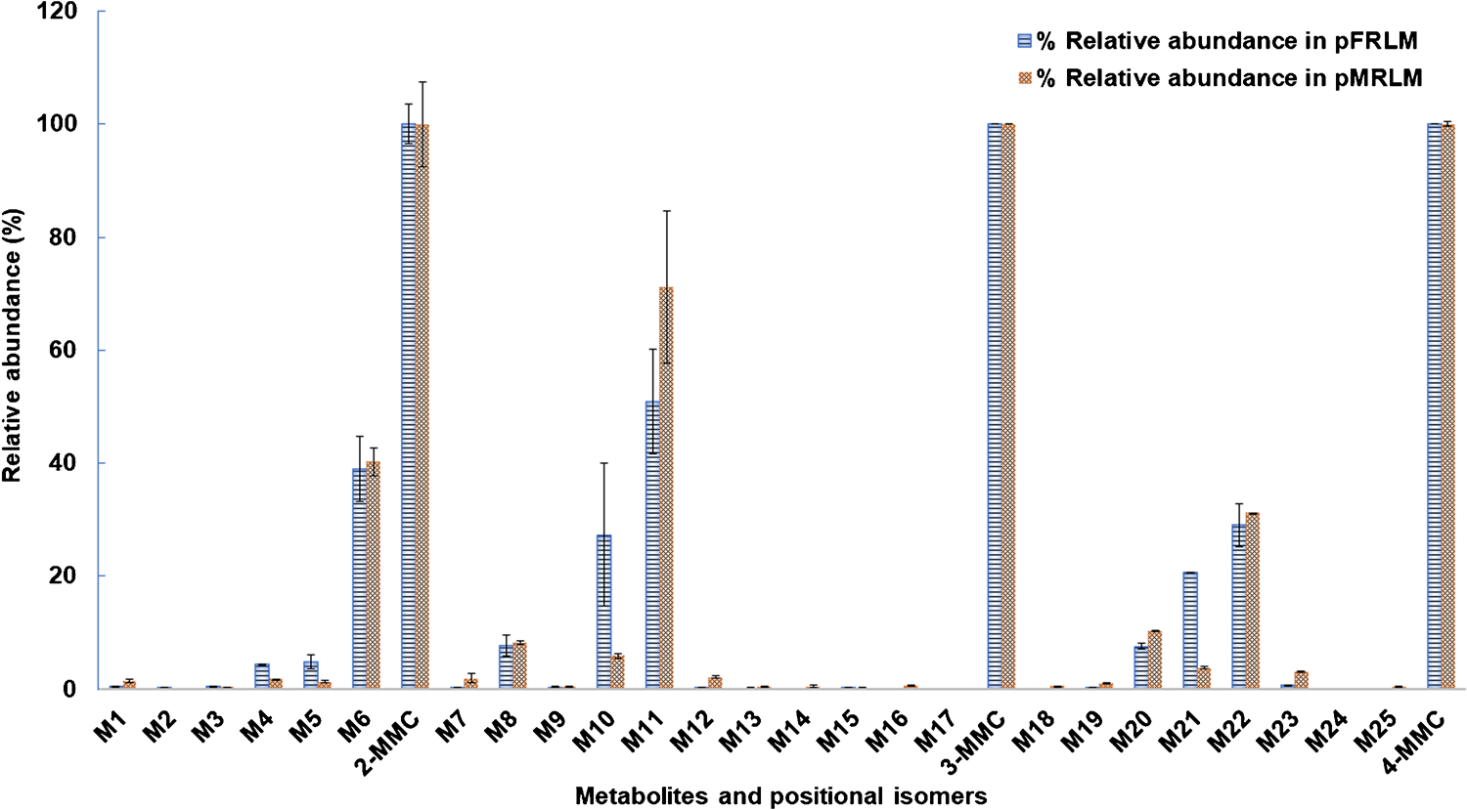
Comparison of the relative abundance observed for the tentatively identified metabolites of MMCs between pFRLM (light blue bars with lined pattern) and pMRLM (orange bars with crossed pattern) incubations. The relative abundance (%) is expressed as the average of the peak area of each metabolite or parent compound (*n* = 2) divided by the average of the peak areas measured for the parent compound in negative controls (*n* = 2) and multiplied by 100. The metabolite numbers correspond to the identified metabolites listed in Table 1. The original data used for this figure are shown in Table S3. Error bars represent the relative standard deviation of the peak area (*n* = 2). Abbreviations: pFRLM, pooled female rat liver microsomal incubations; pMRLM, pooled male rat liver microsomal incubations; MMC, methylmethcathinone

Besides the differences in the metabolites observed (i.e., some metabolites were observed only in pMRLM incubations), sex-specific differences in relative abundance were also observed between pFRLM incubations and pMRLM incubations (see Table S3 and Fig. 8). The phase I positional isomers primary metabolites **M5**, **M10**, and **M21** were present at a relatively higher abundance in pFRLM incubations compared with pMRLM incubations, which underwent the same modifications from their respective parent compound. The phase I primary metabolites can be further metabolized into phase I secondary metabolites, where there were many differences observed between pFRLM incubations and pMRLM incubations. Besides forming a greater number of metabolites compared with pFRLM incubations, pMRLM incubations produced more relatively abundant secondary metabolites via further hydroxylation, demethylation, or hydroxylation with corresponding dehydrogenation. Among these metabolites, the di-hydroxylated metabolites **M12** and **M23** were observed at a relatively higher abundance in pMRLM incubations than pFRLM incubations. Finally, the *N*-demethylated metabolites (**M6**, **M11**, and **M22**) were detected at a relatively similar abundance in both sexes. Since SCs undergo metabolism via a limited number of enzymes, they might compete with each other as substrates for the same enzymes, resulting in metabolic drug-drug interactions [52]. Thus, those sex-specific differences in phase I secondary metabolism might be related with the abundance and genres of enzymes in pMRLM incubations and pFRLM incubations, where the primary metabolites were transformed into secondary metabolites more rapidly in pMRLM incubations than in pFRLM incubations. Certain CYP isozymes responsible for causing the sex-specific differences in the in vitro metabolism of NPS should be further investigated.

This study focused on the metabolic fates of the positional isomers of MMC and investigated possible sex-specific differences in their phase I metabolism. Moreover, because several isoenzymes are involved in drug metabolism and drug-metabolizing enzymes in pFRLM and pMRLM are different in relative abundance and quality, it is essential to investigate to what extent each of these isozymes is involved in drug metabolism, especially for the sex-specific enzymes in rats, such as CYP2A2, CYP2C11, CYP2C13, and CYP3A2, in male rats and the CYP2C12 in female rats.

Strikingly, such differences in metabolite formation and their relative abundance were not observed in pMHLM versus pFHLM incubations (Supplementary Fig. S10 and Supplementary Table S4). As mentioned, the Caucasian-derived liver microsomes used in this study might partially explain this observation. Moreover, the influence of specific isozymes should be studied using liver microsomes with the addition of specific inhibitors in future research to demonstrate exactly which sex-specific isozyme(s) influence the metabolism of MMCs.

## Conclusions and perspectives

This study aimed at identifying the metabolites of MMCs using in vitro metabolic incubations and investigate potential sex-specific differences in MMCs biotransformation. For the first time, the biotransformation of 3-MMC and 2-MMC in liver microsomes was tentatively proposed. The consistent loss of H_2_O followed by the loss of a methyl radical was observed for all MMC positional isomers. Due to the oxo-function at C-1′, MMC positional isomers form a conjugated indole system to maintain stability, thus resulting in the subsequent loss of radicals. This rearrangement and the favourability for the loss of H_2_O for *N*-alkylated synthetic cathinones explain why the product ions of most metabolites preserved the nitrogen atom. The use of isotopic labelling or MS^n^ experiments would help future understanding of the metabolism of MMC positional isomers. In general, the metabolism of 3-MMC showed a more similar in vitro metabolic pattern with 4-MMC than with 2-MMC in pRLM incubations. In addition, 4-OH-MMC and 3-OH-MMC may be used as specific metabolites due to their discriminative retention time, thereby determining the consumption of the MMC positional isomer by detecting 4-OH-MMC and 3-OH-MMC, in addition to the parent compounds.

This study demonstrates that MMCs undergo a similar in vitro metabolic pathway in both rat and human liver microsomes, and that *N*-demethylation appears to be the main route of metabolism in in vitro metabolic incubations. However, the metabolism of MMCs is also dependent on the sex of the in vitro models. Seven metabolites were exclusively discovered in pMRLM incubations, yet this sex-specific difference was not observed in pHLM incubations. In addition, several metabolites were present in different relative abundance between the pMRLM and pFRLM incubations. These results emphasize the need to investigate the biotransformation of xenobiotics by taking sex into consideration to have an integrated overview of their metabolism, and, thus, further improve future NPS risk assessment.

## Supplementary information

Below is the link to the electronic supplementary material.

Supplementary file1 (DOCX 5229 KB)
