# Supplementary material for: In vitro metabolism of cathinone positional isomers: does sex matter?
Source: Anal Bioanal Chem. 2023 Jul 15;415(22):5403–20. doi: 10.1007/s00216-023-04815-3 (PMC10444680; doi:10.1007/s00216-023-04815-3)
Supplement: Supplementary file 1 — Supplementary file1 (DOCX 5229 KB) [file 216_2023_4815_MOESM1_ESM.docx]

***Supplementary Information***

***In vitro metabolism of cathinone positional isomers: does sex matter?***

Peng Che^a, b^, J. Tyler Davidson^c^, Kristina Still^a, b^, Jeroen Kool^a, b^, Isabelle Kohler^a, b, d,∗^

^a^Vrije Universiteit Amsterdam, Amsterdam Institute of Molecular and Life Sciences (AIMMS), Division of BioAnalytical Chemistry, Amsterdam, The Netherlands

^b^Center for Analytical Sciences Amsterdam (CASA), Amsterdam, The Netherlands

^c^Sam Houston State University, Department of Forensic Science, Huntsville, TX, USA

^d^Co van Ledden Hulsebosch Center (CLHC), Amsterdam Center for Forensic Science and Medicine, Amsterdam, The Netherlands

* Corresponding author Dr. Isabelle Kohler, Division of Bioanalytical Chemistry, Amsterdam Institute of Molecular and Life Sciences, Vrije Universiteit Amsterdam, De Boelelaan 1085, 1081 HV Amsterdam, the Netherlands. E-Mail [i.kohler@vu.nl](mailto:i.kohler@vu.nl).

**Contents**

**Table S1** MS and MS/MS experimental conditions used for the analysis of metabolic mixtures using HPLC-MS/MS

**Table S2** MS and MS/MS experimental conditions used for the confirmatory analysis of metabolic mixtures using UHPLC-MS/MS

**Table S3** Peak intensity of phase I metabolites detected in pFRLM and pMRLM incubations and relative abundance of each metabolite (i.e., average of the peak intensity of each metabolite or parent compound relative to the average of the peak intensity of the parent compound in negative controls)

**Table S4** Peak intensity of phase I metabolites detected in pFHLM and pMHLM incubations and relative abundance of each metabolite (i.e., average of the peak intensity of each metabolite or parent compound relative to the average of the peak intensity of the parent compound in negative controls)

**Fig. S1** MS/MS analysis of the metabolite **M7** obtained with the incubation of 3-MMC. (**a**) Extracted ion chromatogram (EIC) of **M7** using HPLC-MS/MS. (**b**) MS/MS spectrum of **M7** including the proposed product ions using HPLC-MS/MS. (**c**) EIC of **M7** using UHPLC-MS/MS. (**d**) MS/MS spectrum of **M7** obtained using the confirmatory analysis with UHPLC-MS/MS

**Fig. S2** MS/MS analysis of the metabolite **M8**-**M10** obtained with the incubation of 3-MMC. (**a**) Extracted ion chromatogram (EIC) of **M8**-**M10** using HPLC-MS/MS. (**b**) MS/MS spectrum of **M8** including the proposed product ions using HPLC-MS/MS. **(c)** MS/MS spectrum of **M9** including the proposed product ions using HPLC-MS/MS. **(d)** MS/MS spectrum of **M10** including the proposed product ions using HPLC-MS/MS. **(e)** EIC of **M8**-**M10** using UHPLC-MS/MS. **(f)** MS/MS spectrum of **M9** obtained using the confirmatory analysis with UHPLC-MS/MS

**Fig. S3** MS/MS analysis of the metabolite **M11** obtained with the incubation of 3-MMC. (**a**) Extracted ion chromatogram of **M11** using HPLC-MS/MS. (**b**) MS/MS spectrum of **M11** including the proposed product ions using HPLC-MS/MS

**Fig. S4** MS/MS analysis of the metabolite **M12** obtained with the incubation of 3-MMC. (**a**) Extracted ion chromatogram of **M12** using HPLC-MS/MS. (**b**) MS/MS spectrum of **M12** including the proposed product ions using HPLC-MS/MS

**Fig. S5** MS/MS analysis of the metabolite **M13** and **M14** obtained with the incubation of 3-MMC. (**a**) Extracted ion chromatogram (EIC) of **M13** and **M14** using HPLC-MS/MS. (**b**) MS/MS spectrum of **M13** including the proposed product ions using HPLC-MS/MS. **(c)** MS/MS spectrum of **M14** including the proposed product ions using HPLC-MS/MS. **(d)** EIC of **M13** and **M14** using UHPLC-MS/MS. **(e)** MS/MS spectrum of **M13** obtained using the confirmatory analysis with UHPLC-MS/MS

**Fig. S6** MS/MS analysis of the metabolite **M115** obtained with the incubation of 3-MMC. (**a**) Extracted ion chromatogram of **M15** using HPLC-MS/MS. (**b**) MS/MS spectrum of **M15** including the proposed product ions using HPLC-MS/MS

**Fig. S7** MS/MS analysis of the metabolite **M16** obtained with the incubation of 3-MMC. (**a**) Extracted ion chromatogram of **M16** using HPLC-MS/MS. (**b**) MS/MS spectrum of **M16** including the proposed product ions using HPLC-MS/MS

**Fig. S8** MS/MS analysis of the metabolite **M17** obtained with the incubation of 3-MMC. (**a**) Extracted ion chromatogram of **M17** using HPLC-MS/MS. (**b**) MS/MS spectrum of **M17** including the proposed product ions using HPLC-MS/MS

**Fig. S9** Comparison of extracted ion chromatograms (EICs) of the discriminative metabolites for 2-MMC and 4-MMC (**M1**, **M18**, **M19**, **M24** and **M25**) measured in pooled male rat liver microsomal (pMRLM) incubations (black EIC traces) which were not detected in pooled female rat liver microsomal (pFRLM) incubations (blue EIC traces). The metabolite numbers correspond to the identified metabolites listed in **Table 1**

**Fig. S10** Comparison of relative abundance observed for the tentatively identified metabolites of MMCs between pFHLM (light blue bars with lined pattern) and pMHLM (orange bars with crossed pattern) incubations. The relative abundance (%) is expressed as the average abundance of metabolites or the parent compound (n=2) divided by the average abundance of the parent compound in negative controls (n=2) and multiplied by 100. The metabolite numbers correspond to the identified metabolites listed in **Table 1**. The original data used for this Figure are shown in **Table S4**. Error bars represent the relative standard deviation of the peak area (n=2). Abbreviations: pFRLM, pooled female rat liver microsomal incubations; pMRLM, pooled male rat liver microsomal incubations; MMC, methylmethcathinones

**Table S1** MS and MS/MS experimental conditions used for the analysis of metabolic mixtures using HPLC-MS/MS

| **MS experimental parameters** | | | | |
| --- | --- | --- | --- | --- |
| Instrument | maXis quadrupole time-of-flight mass spectrometer (Bruker Daltonics, Bremen, Germany) equipped with an ESI source | | | |
| Data acquisition | scan mode | | MS | |
|  | acquisition mode | | line spectra | |
|  | mass range | | *m/z* 50-400 | |
|  | spectral data rate | | 1 Hz (scans/s) | |
|  | absolute threshold | | 25 | |
|  | peak summation width | | 3 pts | |
| ESI parameters | end plate offset voltage | | -500 V | |
|  | capillary voltage | | +4500 V | |
|  | nebulizer gas pressure | | 1.8 bar | |
|  | dry gas flow rate | | 6.0 L/min | |
|  | dry gas temperature | | 200 °C | |
| Parameters of the mass analyzer | transfer funnel RF | | 100 Vpp | |
|  | transfer ISCID energy | | 0 eV | |
|  | multipole RF | | 150 Vpp | |
|  | quadrupole ion energy | | 2 eV | |
|  | quadrupole low mass | | *m/z* 50 | |
|  | collision cell energy | | 5 eV | |
|  | collision RF | | 200 Vpp | |
|  | ion cooler transfer time | | 40 µs | |
|  | ion cooler RF | | 70 Vpp | |
|  | pre pulse storage time | | 10 µs | |
| Time segments | duration of method | | 35.00 min | |
|  | LC eluent directed to waste | | 0.00 - 5.00 min | |
|  | LC eluent directed to ESI-MS | | 5.01 - 35.00 min | |
| Software | compass DataAnalysis® , version 3.2 | | | |
| **MS/MS experimental parameters** | | | | |
| System | maXis® (Bruker Daltonics® GmbH, Bremen, Germany) equipped with an ESI source | | | |
| scan mode | MS/MS-auto | | | |
| precursor-ion-inclusion-list | pos. mode *m/z* 50.00 – 78.00 | | | |
|  | pos. mode *m/z* 79.5.00 – 400.00 | | | |
| precursor ions | 3 x (absolute threshold 2000 cts) | | | |
| smart exclusion | 2 x | | | |
| active exclusion | exclude after 3 spectra, release after 0.50 min | | | |
| isolation mass [*m/z*] | isolation width [*m/z*] | collision energy [eV] | | charge state [z] |
| 100 | 4 | 15 | | 1 |
| 500 | 5 | 25 | | 1 |
| 1000 | 6 | 40 | | 1 |
| Control software | HyStar® 3.2 | | | |

**Table S2** MS and MS/MS experimental conditions used for the analysis of metabolic mixtures using UHPLC-MS/MS

| Instrument | | Zeno quadrupole time-of-flight mass spectrometer (SCIEX, Singapore) equipped with an ESI source | |
| --- | --- | --- | --- |
| parameters | | MS | MS/MS |
| scan mode | | ToF-MS | Information-dependent acquisition (IDA) |
| gas 1 pressure | | 45 psi | |
| gas 2 pressure | | 45 psi | |
| curtain gas pressure | | 30 psi | |
| source temperature | | 500 °C | |
| ion spray voltage | | 5500 V | |
| declustering potential | | 50 V | |
| collision energy | | 10 eV | |
| collision-activated dissociation (CAD) gas pressure | | 7 psi | |
| IDA criteria | | small molecules | |
| maximum candidate ion | | 6 | |
| intensity threshold | | 100 cps | |
| exclusion time | | 6 s after 2 occurrences | |
| start mass | | *m/z* 100 | *m/z* 50 |
| stop mass | | *m/z* 500 | *m/z* 500 |
| electron transfer coefficient  (ETC) | | NA | 100 |
| Zeno trap | | NA | ON |
| accumulation time | | 0.25 s | 0.10 s |
| time bins to sum | | 8 | 12 |
| Collision induced dissociation (CID) mode | Collision energy | NA | 35 eV |
|  | Collision energy spread | NA | 15 eV |

**Table S3** MS Peak intensity of phase I metabolites detected in pFRLM and pMRLM incubations and relative abundance of each metabolite (i.e., average of the peak intensity of each metabolite or parent compound relative to the average of the peak intensity of the parent compound in negative controls).

| Positional isomer | Metabolites | Intensity in pFRLM incubations | | | | | Intensity in pMRLM incubations | | | | |  |
| --- | --- | --- | --- | --- | --- | --- | --- | --- | --- | --- | --- | --- |
|  |  | pFRLM, analysis #1 | pFRLM, analysis #2 | Average (n=2) | RSD (%, n=2) | Relative abundance (%) | pMRLM, analysis #1 | pMRLM, analysis #2 | Average (n=2) | RSD (%, n=2) | Relative abundance (%) |  |
| 2-MMC | M1 | 17996 | 22839 | 20417.5 | 11.9 | 0.7 | 123828 | 80206 | 102017 | 21.4 | 1.5 |  |
|  | M2 | 15501 | 13142 | 14321.5 | 8.2 | 0.5 | 11338 | 11982 | 11660 | 2.8 | 0.2 |  |
|  | M3 | 21908 | 23994 | 22951 | 4.5 | 0.8 | 21717 | 24543 | 23130 | 6.1 | 0.4 |  |
|  | M4 | 192891 | 196666 | 194778.5 | 1.0 | 6.7 | 124268 | 121951 | 123109.5 | 0.9 | 1.8 |  |
|  | M5 | 272854 | 163296 | 218075 | 25.1 | 7.5 | 101514 | 74011 | 87762.5 | 15.7 | 1.3 |  |
|  | M6 | 1984486 | 1468036 | 1726261 | 15.0 | 59.0 | 2533151 | 2859281 | 2696216 | 6.0 | 40.3 |  |
|  | 2-MMC | 4272895 | 4582210 | 4427552.5 | 3.5 | 100.0 | 6192442 | 7203069 | 6697755.5 | 7.5 | 100.0 |  |
| 3-MMC | M7 | 30611 | 28886 | 29748.5 | 0.0 | 0.4 | 203411 | 76808 | 140109.5 | 45.2 | 1.9 |  |
|  | M8 | 696378 | 426361 | 561369.5 | 24.1 | 7.8 | 578057 | 619921 | 598989 | 3.5 | 8.3 |  |
|  | M9 | 40888 | 22297 | 31592.5 | 29.4 | 0.4 | 33401 | 28282 | 30841.5 | 8.3 | 0.4 |  |
|  | M10 | 1062277 | 2878753 | 1970515 | 46.1 | 27.4 | 401667 | 451269 | 426468 | 5.8 | 5.9 |  |
|  | M11 | 4331722 | 2997227 | 3664474.5 | 18.2 | 50.9 | 6092438 | 4150894 | 5121666 | 19.0 | 71.1 |  |
|  | M12 | 11406 | 25092 | 18249 | 37.5 | 0.3 | 167899 | 136651 | 152275 | 10.3 | 2.1 |  |
|  | M13 | 18059 | 14763 | 16411 | 10.0 | 0.2 | 32596 | 38461 | 35528.5 | 8.3 | 0.5 |  |
|  | M14 | 0 | 0 | 0 | 0.0 | 0.0 | 50193 | 20394 | 35293.5 | 42.2 | 0.5 |  |
|  | M15 | 20453 | 15533 | 17993 | 13.7 | 0.3 | 27087 | 16336 | 21711.5 | 24.8 | 0.3 |  |
|  | M16 | 0 | 0 | 0 | 0.0 | 0.0 | 51631 | 39075 | 45353 | 13.8 | 0.6 |  |
|  | M17 | 2497 | 3847 | 3172 | 21.3 | 16.7 | 5293 | 4773 | 5033 | 5.2 | 0.1 |  |
|  | 3-MMC | 7203839 | 7203839 | 7203839 | 0.0 | 100.0 | 7203839 | 7203839 | 7203839 | 0.0 | 100.0 |  |
| 4-MMC | M18 | 0 | 0 | 0 | 0.0 | 0.0 | 41045 | 42445 | 41745 | 1.7 | 0.6 |  |
|  | M19 | 26037 | 11972 | 19004.5 | 37.0 | 0.3 | 77798 | 72478 | 75138 | 3.5 | 1.1 |  |
|  | M20 | 490112 | 610180 | 550146 | 10.9 | 7.6 | 743226 | 720307 | 731766.5 | 1.6 | 10.3 |  |
|  | M21 | 1495493 | 1491441 | 1493467 | 0.1 | 20.7 | 242604 | 301837 | 272220.5 | 10.9 | 3.8 |  |
|  | M22 | 1540916 | 2649220 | 2095068 | 26.5 | 29.1 | 2233905 | 2228198 | 2231051.5 | 0.1 | 31.3 |  |
|  | M23 | 55567 | 44648 | 50107.5 | 10.9 | 0.7 | 199407 | 236218 | 217812.5 | 8.5 | 3.1 |  |
|  | M24 | 0 | 0 | 0 | 0.0 | 0.0 | 10701 | 7628 | 9164.5 | 16.8 | 0.1 |  |
|  | M25 | 0 | 0 | 0 | 0.0 | 0.0 | 32003 | 28175 | 30089 | 6.4 | 0.4 |  |
|  | 4-MMC | 7203839 | 7203839 | 7203839 | 0.0 | 100.0 | 7084401 | 7193494 | 7138947.5 | 0.8 | 100.0 |  |

Abbreviations MMC, methylmethcathinone; pMRLM, pooled male rat liver microsomes; pFRLM, pooled female rat liver microsomes. The metabolite numbers correspond to the identified metabolites listed in **Table 1.**

**Table S4** MS Peak intensity of phase I metabolites detected in pFHLM and pMHLM incubations and relative abundance of each metabolite (i.e., average of the peak intensity of each metabolite or parent compound relative to the average of the peak intensity of the parent compound in negative controls)

| Positional isomer | Metabolites | Intensity in pFHLM incubations | | | | | Intensity in pMHLM incubations | | | | |  |
| --- | --- | --- | --- | --- | --- | --- | --- | --- | --- | --- | --- | --- |
|  |  | pFHLM, analysis #1 | pFHLM, analysis #2 | Average (n=2) | RSD (%, n=2) | Relative abundance (%) | pMHLM, analysis #1 | pMHLM, analysis #2 | Average (n=2) | RSD (%, n=2) | Relative abundance (%) | |
| 2-MMC | M1 | 0 | 0 | 0 | NA | 0.0 | 0 | 0 | 0 | NA | 0.0 | |
|  | M2 | 0 | 0 | 0 | NA | 0.0 | 0 | 0 | 0 | NA | 0.0 | |
|  | M3 | 0 | 0 | 0 | NA | 0.0 | 0 | 0 | 0 | NA | 0.0 | |
|  | M4 | 0 | 0 | 0 | NA | 0.0 | 0 | 0 | 0 | NA | 0.0 | |
|  | M5 | 219540 | 253387 | 236464 | 7.2 | 3.3 | 208544 | 323472 | 266008 | 21.6 | 3.7 | |
|  | M6 | 1348098 | 1403973 | 1376036 | 2.0 | 19.2 | 1050548 | 1403973 | 1227261 | 14.4 | 17.0 | |
|  | 2-MMC | 7135011 | 7203839 | 7169425 | 0.5 | 100.0 | 7203839 | 7198810 | 7201325 | 0.0 | 100.0 | |
| 3-MMC | M7 | 0 | 0 | 0 | NA | 0.0 | 0 | 0 | 0 | NA | 0.0 | |
|  | M8 | 522594 | 853012 | 687803 | 24.0 | 9.6 | 775754 | 780332 | 778043 | 0.3 | 10.8 | |
|  | M9 | 0 | 0 | 0 | NA | 0.0 | 0 | 0 | 0 | NA | 0.0 | |
|  | M10 | 382289 | 224976 | 303633 | 25.9 | 4.2 | 424294 | 489469 | 456882 | 7.1 | 6.4 | |
|  | M11 | 528256 | 832493 | 680375 | 22.4 | 9.4 | 892826 | 871449 | 882138 | 1.2 | 12.3 | |
|  | M12 | 0 | 0 | 0 | NA | 0.0 | 0 | 0 | 0 | NA | 0.0 | |
|  | M13 | 0 | 0 | 0 | NA | 0.0 | 0 | 0 | 0 | NA | 0.0 | |
|  | M14 | 0 | 0 | 0 | NA | 0.0 | 0 | 0 | 0 | NA | 0.0 | |
|  | M15 | 0 | 0 | 0 | NA | 0.0 | 0 | 0 | 0 | NA | 0.0 | |
|  | M16 | 0 | 0 | 0 | NA | 0.0 | 0 | 0 | 0 | NA | 0.0 | |
|  | M17 | 0 | 0 | 0 | NA | 0.0 | 0 | 0 | 0 | NA | 0.0 | |
|  | 3-MMC | 7203839 | 7203839 | 7203839 | 0.0 | 100.0 | 7188079 | 7203839 | 7195959 | 0.1 | 100.0 | |
| 4-MMC | M18 | 0 | 0 | 0 | NA | 0.0 | 0 | 0 | 0 | NA | 0.0 | |
|  | M19 | 0 | 0 | 0 | NA | 0.0 | 0 | 0 | 0 | NA | 0.0 | |
|  | M20 | 1419456 | 1432682 | 1426069 | 0.5 | 19.8 | 1404563 | 1224572 | 1314568 | 6.9 | 18.3 | |
|  | M21 | 382023 | 318466 | 350245 | 9.1 | 4.9 | 576030 | 1216292 | 896161 | 35.7 | 12.4 | |
|  | M22 | 485237 | 333963 | 409600 | 18.5 | 5.7 | 549429 | 879556 | 714493 | 23.1 | 9.9 | |
|  | M23 | 0 | 0 | 0 | NA | 0.0 | 0 | 0 | 0 | NA | 0.0 | |
|  | M24 | 0 | 0 | 0 | NA | 0.0 | 0 | 0 | 0 | NA | 0.0 | |
|  | M25 | 0 | 0 | 0 | NA | 0.0 | 0 | 0 | 0 | NA | 0.0 | |
|  | 4-MMC | 7203839 | 7203839 | 7203839 | 0.0 | 100.0 | 7203839 | 7203839 | 7203839 | 0.00 | 100.0 | |

Abbreviations N.A., not applicable; MMC, methylmethcathinone; pMHLM, pooled male human liver microsomes; pFHLM, pooled female human liver microsomes. Metabolite numbers correspond to the identified metabolites listed in **Table 1**


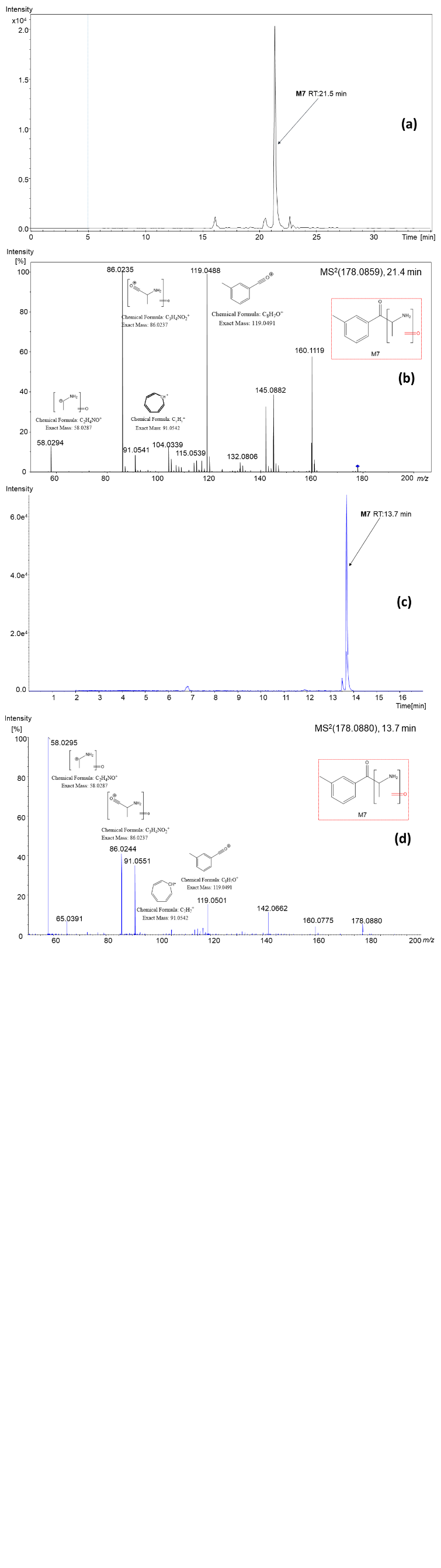


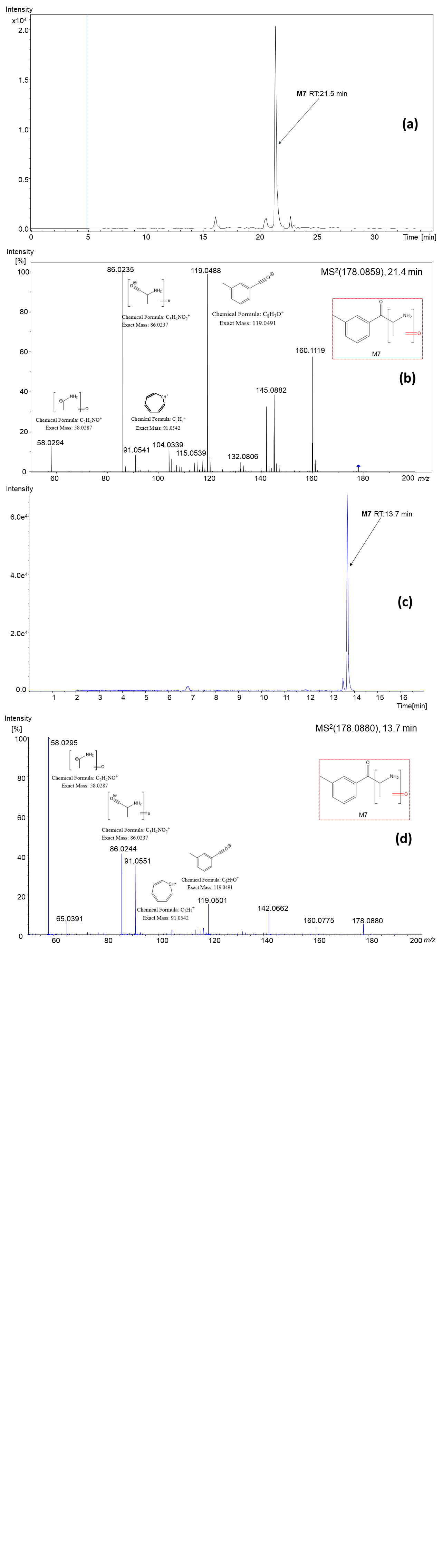


**Fig. S1** MS/MS analysis of the metabolite **M7** obtained with the incubation of 3-MMC. (**a**) Extracted ion chromatogram (EIC) of **M7** using HPLC-MS/MS. (**b**) MS/MS spectrum of **M7** including the proposed product ions using HPLC-MS/MS. **(c)** EIC of **M7** using UHPLC-MS/MS. **(d)** MS/MS spectrum of **M7** obtained using the confirmatory analysis with UHPLC-MS/MS.


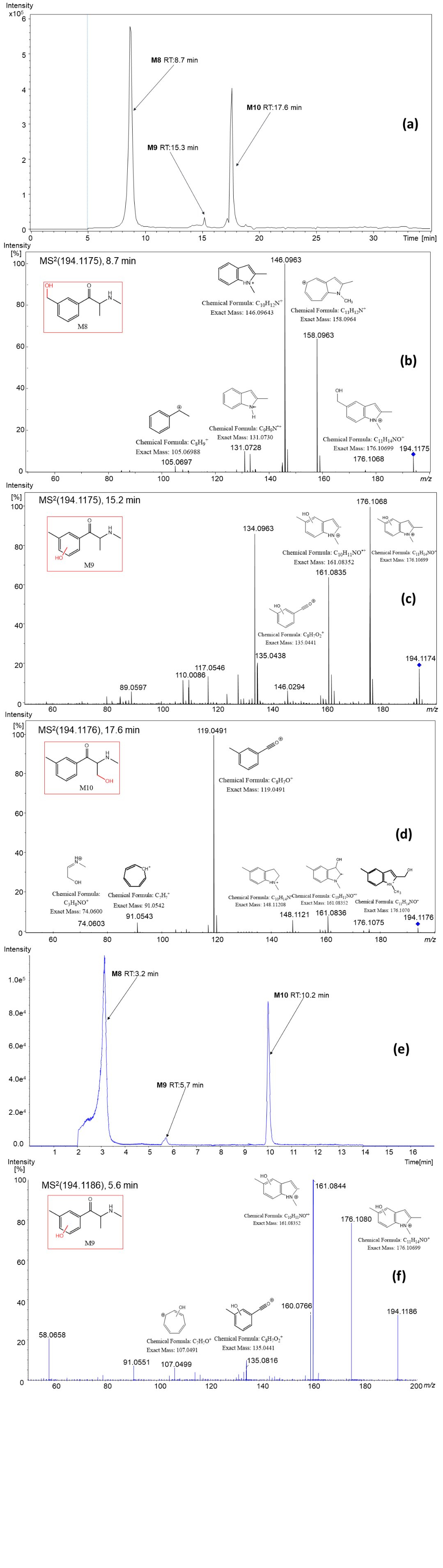

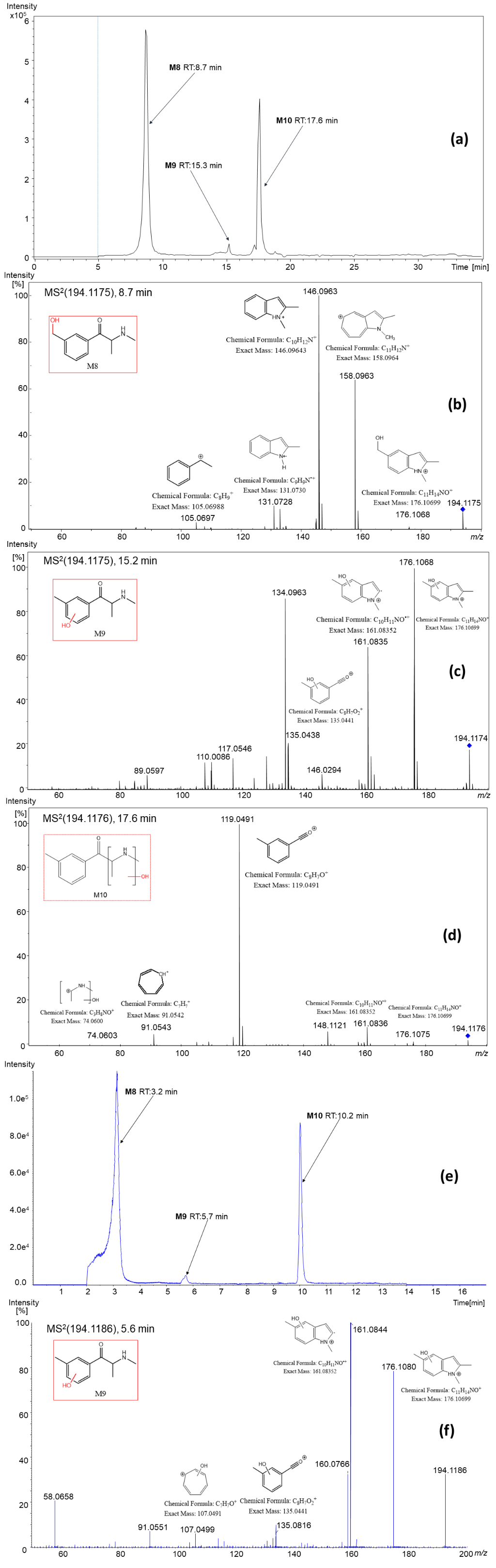


**Fig. S2** MS/MS analysis of the metabolite **M8**-**M10** obtained with the incubation of 3-MMC. (**a**) Extracted ion chromatogram (EIC) of **M8**-**M10** using HPLC-MS/MS. (**b**) MS/MS spectrum of **M8** including the proposed product ions using HPLC-MS/MS. **(c)** MS/MS spectrum of **M9** including the proposed product ions using HPLC-MS/MS. **(d)** MS/MS spectrum of **M10** including the proposed product ions using HPLC-MS/MS. **(e)** EIC of **M8**-**M10** using UHPLC-MS/MS. **(f)** MS/MS spectrum of **M9** obtained using the confirmatory analysis with UHPLC-MS/MS.


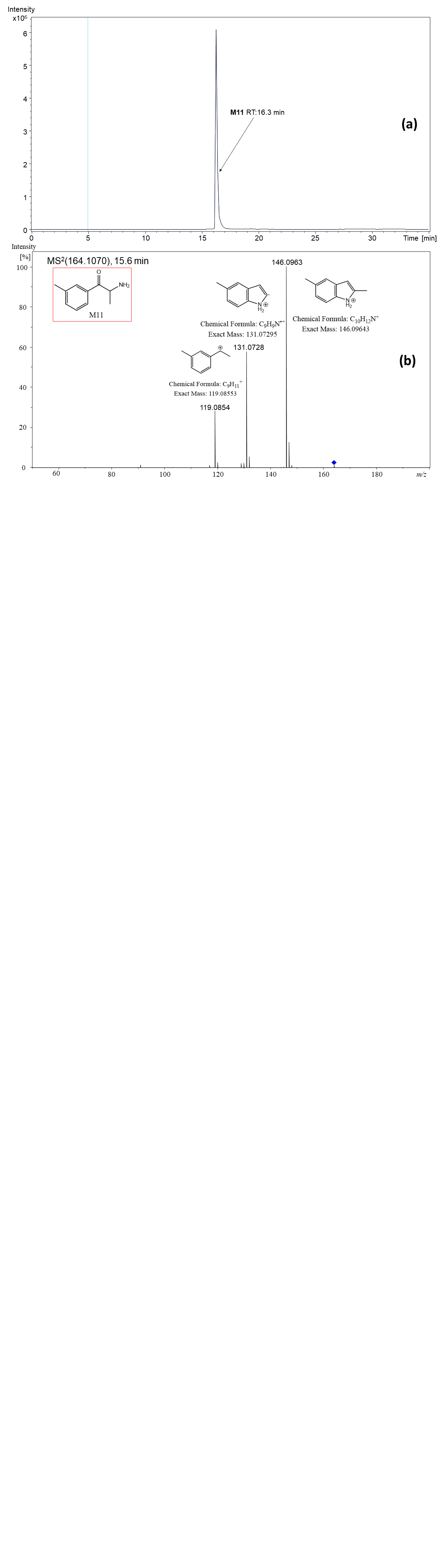


**Fig. S3** MS/MS analysis of the metabolite **M11** obtained with the incubation of 3-MMC. (**a**) Extracted ion chromatogram of **M11** using HPLC-MS/MS. (**b**) MS/MS spectrum of **M11** including the proposed product ions using HPLC-MS/MS.


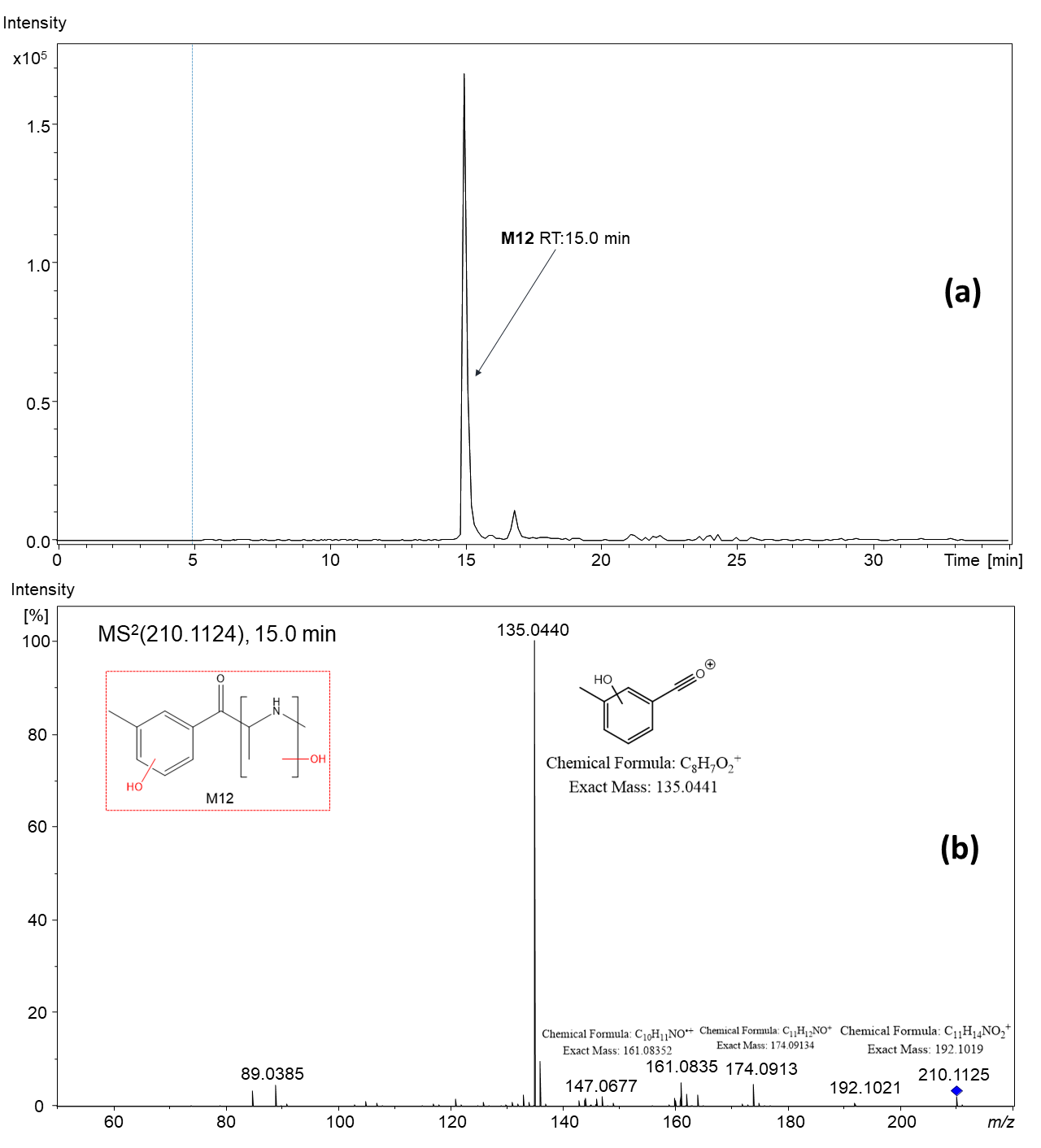


**Fig. S4** MS/MS analysis of the metabolite **M12** obtained with the incubation of 3-MMC. (**a**) Extracted ion chromatogram of **M12** using HPLC-MS/MS. (**b**) MS/MS spectrum of **M12** including the proposed product ions using HPLC-MS/MS.


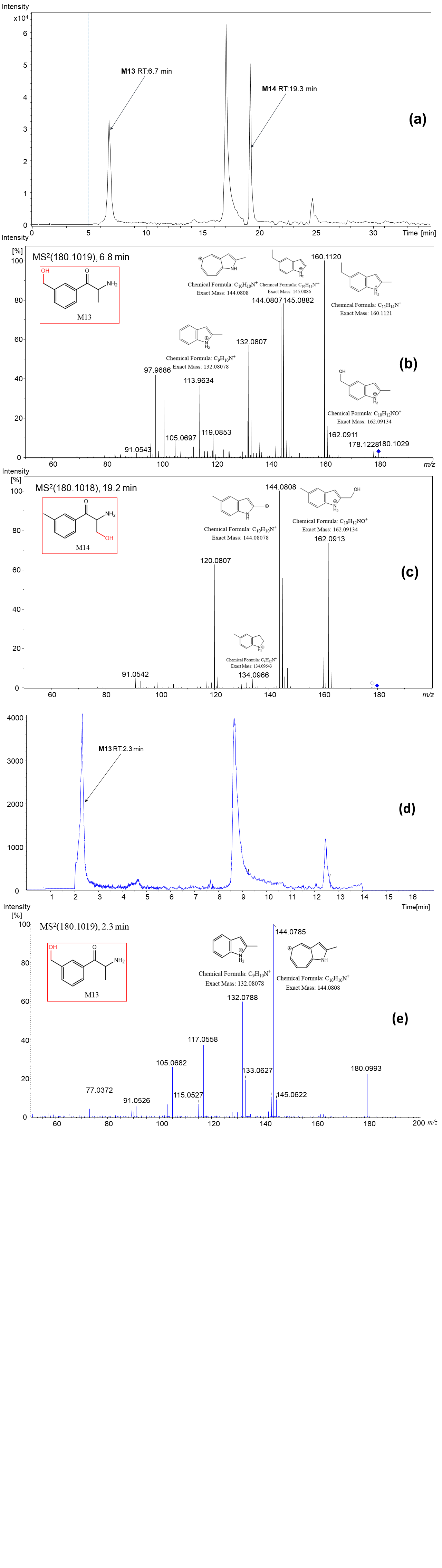


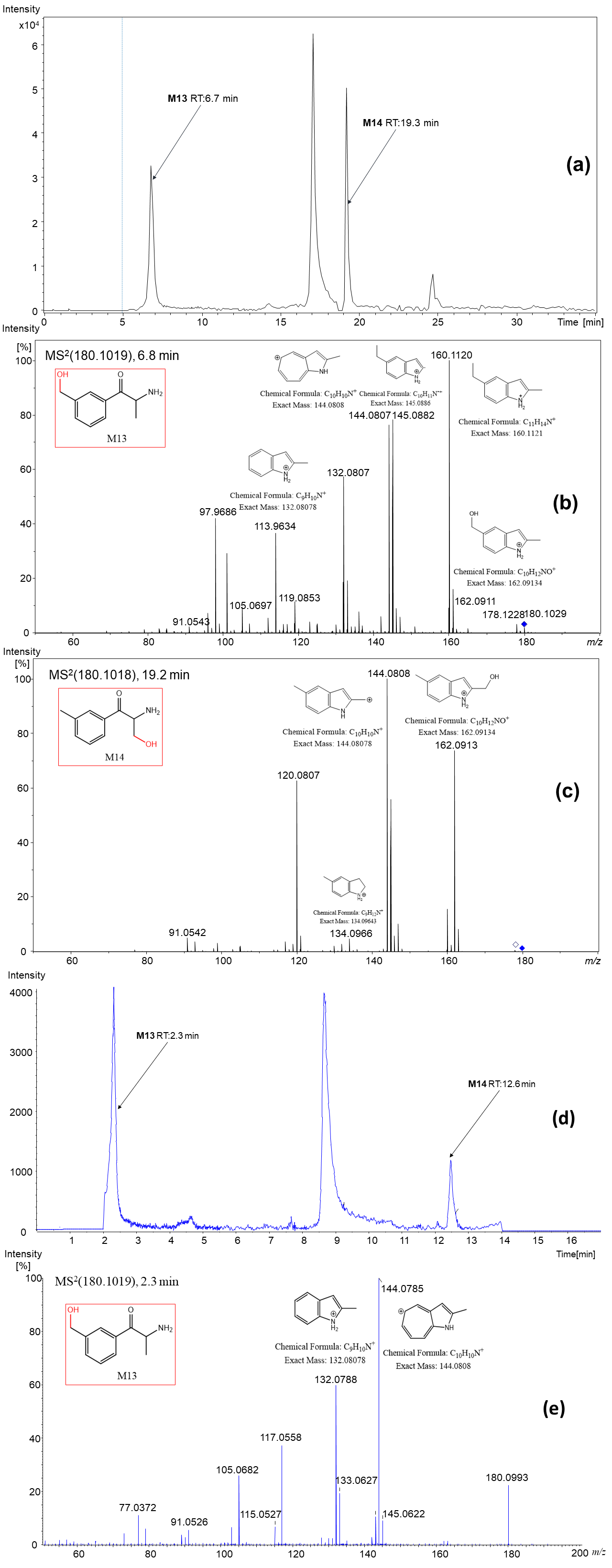


**Fig. S5** MS/MS analysis of the metabolite **M13** and **M14** obtained with the incubation of 3-MMC. (**a**) Extracted ion chromatogram (EIC) of **M13** and **M14** using HPLC-MS/MS. (**b**) MS/MS spectrum of **M13** including the proposed product ions using HPLC-MS/MS. **(c)** MS/MS spectrum of **M14** including the proposed product ions using HPLC-MS/MS. **(d)** EIC of **M13** and **M14** using UHPLC-MS/MS. **(e)** MS/MS spectrum of **M13** obtained using the confirmatory analysis with UHPLC-MS/MS.


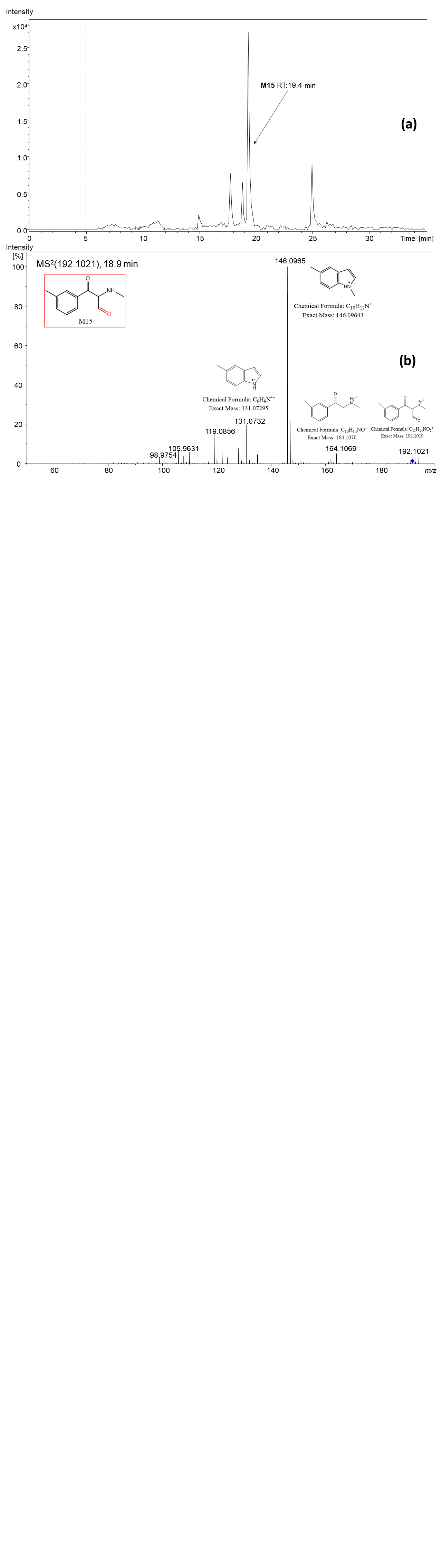


**Fig. S6** MS/MS analysis of the metabolite **M15** obtained with the incubation of 3-MMC. (**a**) Extracted ion chromatogram of **M15** using HPLC-MS/MS. (**b**) MS/MS spectrum of **M15** including the proposed product ions using HPLC-MS/MS.


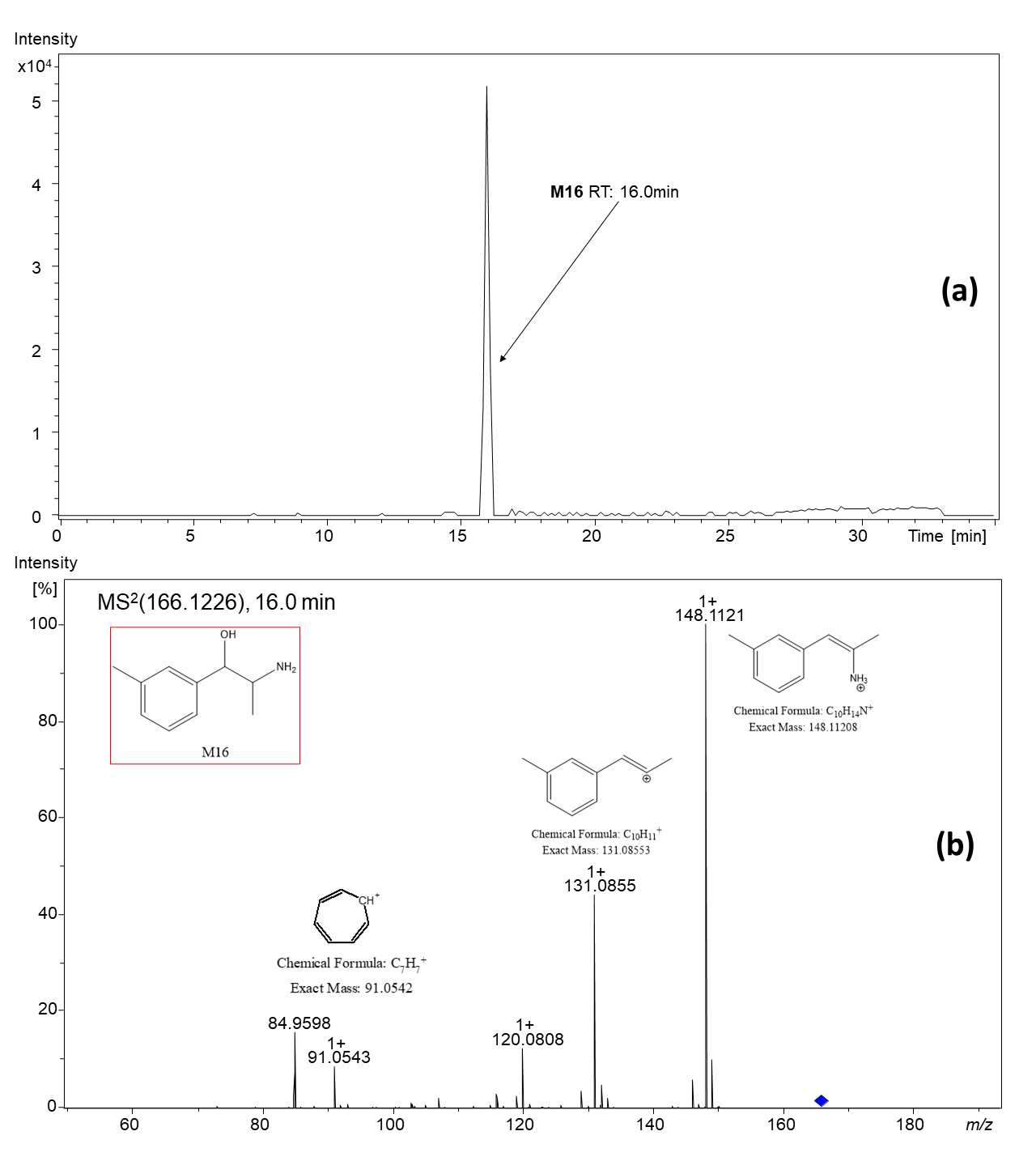


**Fig. S7** MS/MS analysis of the metabolite **M16** obtained with the incubation of 3-MMC. (**a**) Extracted ion chromatogram of **M16** using HPLC-MS/MS. (**b**) MS/MS spectrum of **M16** including the proposed product ions using HPLC-MS/MS.


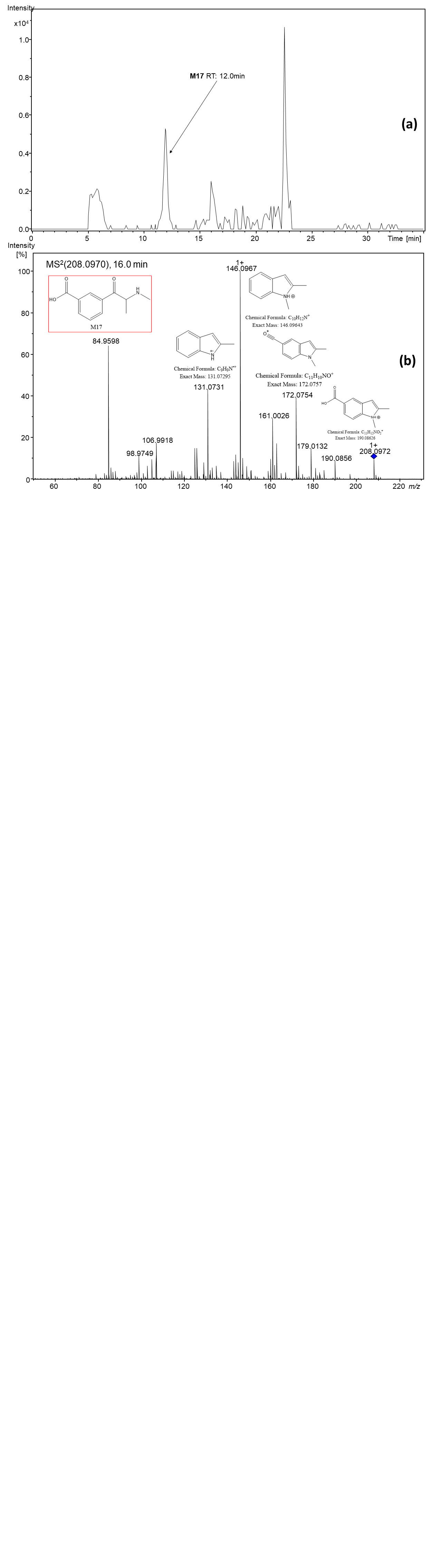


**Fig. S8** MS/MS analysis of the metabolite **M17** obtained with the incubation of 3-MMC. (**a**) Extracted ion chromatogram of **M17** using HPLC-MS/MS. (**b**) MS/MS spectrum of **M17** including the proposed product ions using HPLC-MS/MS.


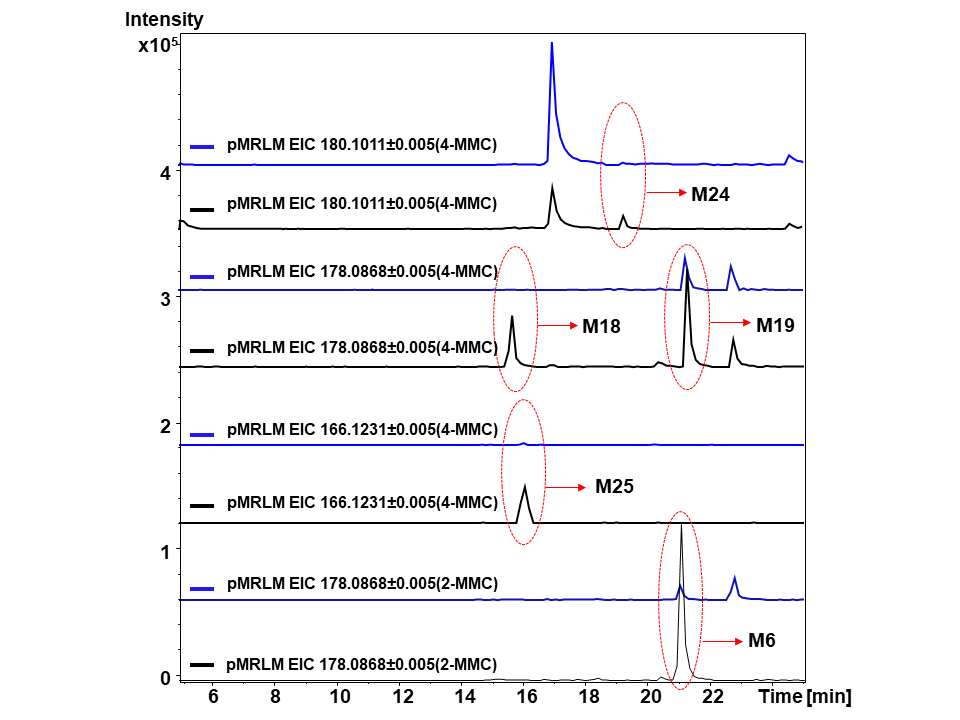


**Fig. S9** Comparison of extracted ion chromatograms (EICs) of the discriminative metabolites for 2-MMC and 4-MMC (**M1**, **M18**, **M19**, **M24** and **M25**) measured in pooled male rat liver microsomal (pMRLM) incubations (black EIC traces) which were not detected in pooled female rat liver microsomal (pFRLM) incubations (blue EIC traces). The metabolite numbers correspond to the identified metabolites listed in **Table 1.**


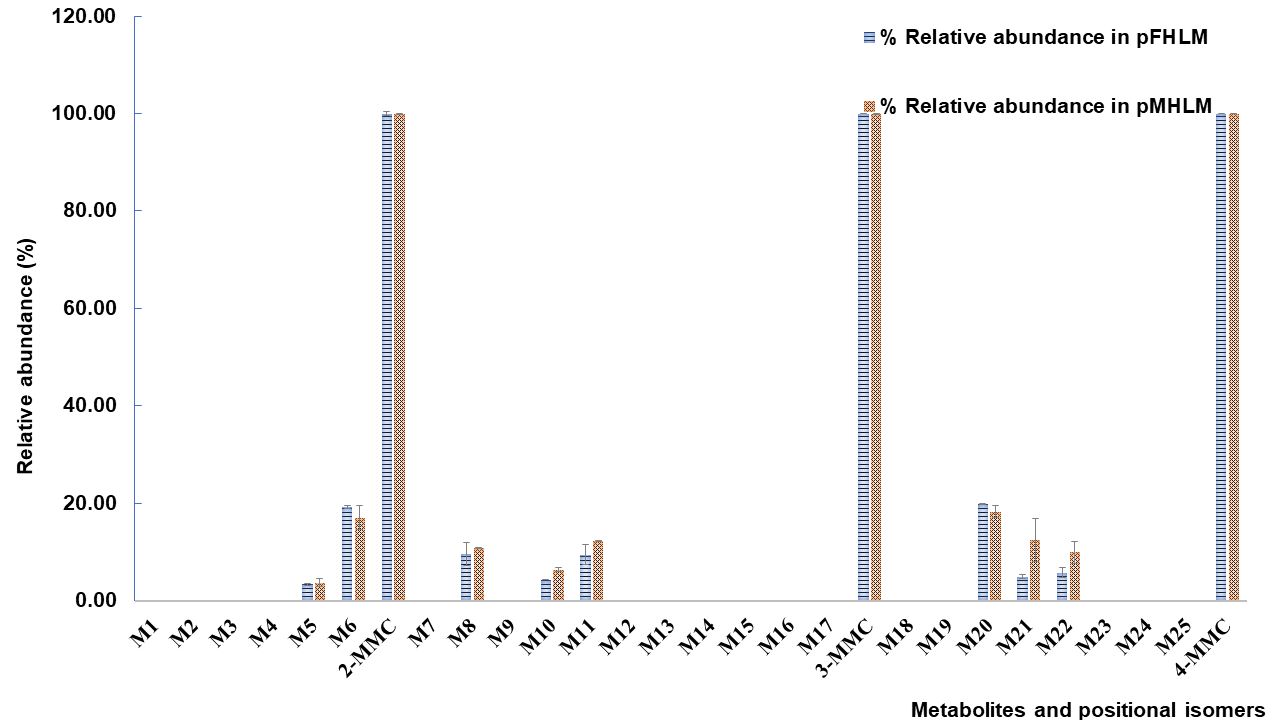
**Fig. S10** Comparison of relative abundance observed for the tentatively identified metabolites of MMCs between pFHLM (light blue bars with lined pattern) and pMHLM (orange bars with crossed pattern) incubations. The relative abundance (%) is expressed as the average abundance of metabolites or the parent compound (n=2) divided by the average abundance of the parent compound in negative controls (n=2) and multiplied by 100. The metabolite numbers correspond to the identified metabolites listed in **Table 1**. The original data used for this Figure are shown in **Table S4**. Error bars represent the relative standard deviation of the peak area (n=2). Abbreviations: pFRLM, pooled female rat liver microsomal incubations; pMRLM, pooled male rat liver microsomal incubations; MMC, methylmethcathinone.
